# In vivo imaging of inflammation and oxidative stress in a nonhuman primate model of cardiac sympathetic neurodegeneration

**DOI:** 10.1038/s41531-018-0057-1

**Published:** 2018-07-13

**Authors:** Jeanette M. Metzger, Colleen F. Moore, Carissa A. Boettcher, Kevin G. Brunner, Rachel A. Fleddermann, Helen N. Matsoff, Henry A. Resnikoff, Viktoriya Bondarenko, Timothy J. Kamp, Timothy A. Hacker, Todd E. Barnhart, Patrick J. Lao, Bradley T. Christian, R. Jerry Nickles, Catherine L. Gallagher, James E. Holden, Marina E. Emborg

**Affiliations:** 1 Preclinical Parkinson’s Research Program, Wisconsin National Primate Research Center, University of Wisconsin–Madison, Madison, WI USA; 2 Cellular and Molecular Pathology Graduate Program, University of Wisconsin–Madison, Madison, WI USA; 3 Department of Psychology, University of Wisconsin–Madison, Madison, WI USA; 4 Department of Medicine, University of Wisconsin–Madison, Madison, WI USA; 5 Department of Medical Physics, University of Wisconsin–Madison, Madison, WI USA; 6 Department of Neurology, University of Wisconsin–Madison, Madison, WI USA

## Abstract

Loss of cardiac postganglionic sympathetic innervation is a characteristic pathology of Parkinson’s disease (PD). It progresses over time independently of motor symptoms and is not responsive to typical anti-parkinsonian therapies. Cardiac sympathetic neurodegeneration can be mimicked in animals using systemic dosing of the neurotoxin 6-hydroxydopamine (6-OHDA). As in PD, 6-OHDA-induced neuronal loss is associated with increased inflammation and oxidative stress. To assess the feasibility of detecting changes over time in cardiac catecholaminergic innervation, inflammation, and oxidative stress, myocardial positron emission tomography with the radioligands [^11^C]*meta*-hydroxyephedrine (MHED), [^11^C]PBR28 (PBR28), and [^61^Cu]diacetyl-*bis*(*N*(4))-methylthiosemicarbazone (ATSM) was performed in 6-OHDA-intoxicated adult, male rhesus macaques (*n* = 10; 50 mg/kg i.v.). The peroxisome proliferator-activated receptor gamma (PPARγ) agonist pioglitazone, which is known to have anti-inflammatory and anti-oxidative stress properties, was administered to five animals (5 mg/kg, PO); the other five were placebo-treated. One week after 6-OHDA, cardiac MHED uptake was significantly reduced in both groups (placebo, 86% decrease; pioglitazone, 82%); PBR28 and ATSM uptake increased in both groups but were attenuated in pioglitazone-treated animals (PBR28 Treatment × Level ANOVA *p* < 0.002; ATSM Mann–Whitney *p* = 0.032). At 12 weeks, partial recovery of MHED uptake was significantly greater in the pioglitazone-treated group, dependent on left ventricle circumferential region and axial level (Treatment × Region × Level ANOVA *p* = 0.034); 12-week MHED uptake significantly correlated with tyrosine hydroxylase immunoreactivity across cardiac anatomy (*p* < 0.000002). PBR28 and ATSM uptake returned to baseline levels by 12 weeks. These radioligands thus hold potential as in vivo biomarkers of mechanisms of cardiac neurodegeneration and neuroprotection.

## Cardiac neurodegeneration: Visualizing the damage

Three cardiac nerve loss biomarkers enable the visualization of cardiac neurodegeneration and response to neuroprotective treatment. The loss of sympathetic cardiac innervation in patients with PD causes symptoms such as postural hypotension, arrhythmia, and fatigue that do not respond to anti-parkinsonian medications. Marina Emborg and colleagues at University of Wisconsin–Madison, USA, used positron emission tomography with three radioligands to image changes in cardiac innervation, oxidative stress and inflammation in monkeys during neurotoxin-induced PD-like cardiac neurodegeneration. They were able to visualize the recruitment of inflammatory cells and increased production of reactive oxygen species during neurodegeneration as well as observe improvements in response to pioglitazone, a drug that has previously been shown to have neuroprotective effects in animal models of PD. These radioligands could be useful imaging biomarkers of cardiac nerve loss progression in patients with PD or cardiovascular disease.

## Introduction

Sixty percent of Parkinson’s disease (PD) patients are estimated to have loss of postganglionic sympathetic innervation to the heart at time of diagnosis.^1^ This degeneration progresses independent of motor symptoms,^2,3^ eventually affecting an estimated 100% of PD patients.^4^ Associated cardiovascular symptoms include fatigue and lowered cardiac contractility during exercise.^5,6^ Many PD patients also have decreased circulating catecholamines and arterial baroreflex failure, a combination known as cardiac dysautonomia,^6^ which can cause dizziness, syncope, and increased risk of falls and injury.^6,7^ Cardiac sympathetic nerve loss is reliably identified by in vivo imaging with sympathomimetic radioligands.^8^ Imaging evidence of decreased cardiac postganglionic sympathetic innervation is now a supportive criterion for clinical diagnosis of PD^9^ and has recently been suggested to predict PD diagnosis in at-risk individuals.^10^ Symptoms of cardiac denervation and dysautonomia are not alleviated by anti-parkinsonian therapies and cannot be prevented or slowed. Although the etiology of PD remains unknown, accumulated evidence shows that inflammation and oxidative stress play a role in PD neurodegeneration and could be targeted to induce neuroprotection.^11,12^

Systemic administration of the catecholaminergic neurotoxin 6-hydroxydopamine (6-OHDA) induces PD-like cardiac sympathetic denervation in nonhuman primates,^13,14^ which can be detected by in vivo imaging with the catecholamine analog [^11^C]*meta*-hydroxyephedrine (MHED).^14^ 6-OHDA is uptaken by catecholamine transporters and autoxidizes, rapidly producing reactive oxygen species and recruiting proinflammatory immune cells. It also compromises mitochondrial complex I activity, further contributing to reactive oxygen species burden.^15^ Similar to PD neurodegeneration, 6-OHDA-induced catecholaminergic neuronal loss is associated with increased oxidative stress and inflammation.

Positron emission tomography (PET) with a new generation of radioligands presents an opportunity for in vivo assessment of the immune response and free radical production during neurodegeneration, as well as for target validation of candidate treatments. [^11^C]PBR28 is a second-generation 18-kDa translocator protein (TSPO) ligand.^16,17^ TSPO binds cholesterol on the outer mitochondrial membrane; it is present in macrophages and, upon activation, its expression significantly increases.^18^ PET with PBR28 identified inflammation in the central nervous system of patients with PD.^19^ Imaging with TSPO ligands has been used to assess cardiovascular pathologies in humans and in rodent models.^16,20,21^ [^61^Cu]Diacetyl-*bis*(*N*(4))-methylthiosemicarbazone (ATSM) allows visualization of oxidative stress via bioreductive trapping of the radioactive copper.^22,23^ PET with ATSM detected oxidative stress in vivo in the striatum of PD patients;^24^ this tracer has also been applied to myocardial ischemia in vivo in humans^25^ and ex vivo in rodents.^23^

In the present study, we used PET with MHED, PBR28, and ATSM to assess cardiac changes over time in the systemic 6-OHDA nonhuman primate model. Based on our previous investigations,^14^ we hypothesized that 6-OHDA-induced cardiac sympathetic loss would be associated with increased inflammation and oxidative stress at 1 week after 6-OHDA neurointoxication, and that these neurodegenerative mechanisms would resolve by 3 months. To further assess the sensitivity of the imaging tools, we treated half the animals with daily oral dosing of pioglitazone, a peroxisome proliferator-activated receptor gamma (PPARγ) agonist known to modulate the expression of genes involved in the cellular response to oxidative stress and inflammation.^26^ Our overall aim was to develop methods for in vivo visualization of mechanisms of neurodegeneration and target validation of potential therapeutics for PD cardiac dysautonomia.

## Results

### Overall animal health

**Fig. 1:**
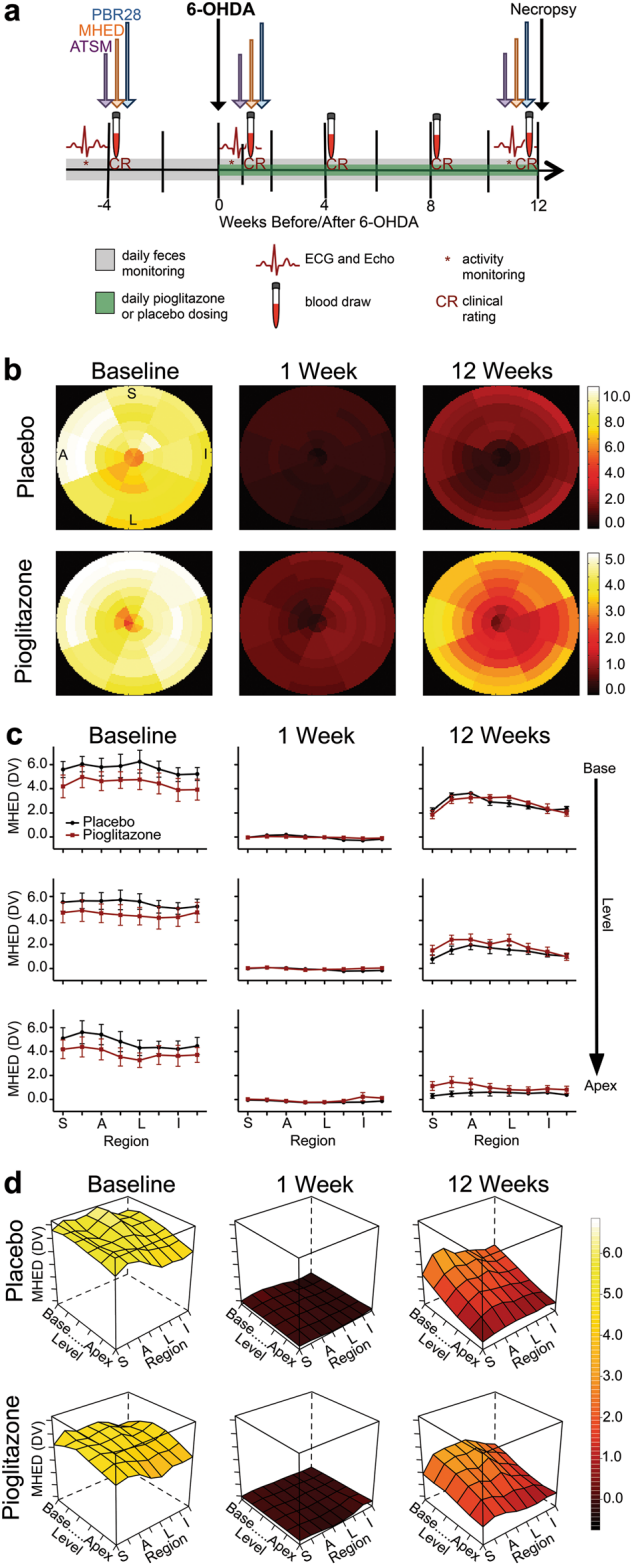
Experimental timeline and [^11^C]MHED PET. **a** At least 1 week after baseline PET scans, ten adult rhesus macaques underwent systemic administration of 6-OHDA (50 mg/kg i.v.). At 24 h after 6-OHDA, animals were blindly, randomly assigned to either daily oral dosing of placebo (*n* = 5) or pioglitazone (*n* = 5; 5 mg/kg). **b** Cardiac distribution volume (DV) maps of MHED uptake at baseline, 1, and 12 weeks after 6-OHDA in representative placebo- and pioglitazone-treated monkeys (apex of heart at center, base of left ventricle at edge). Letters indicate region orientation (S septal, I inferior, L lateral, A anterior). Increased recovery of MHED uptake from 1 week to 12 weeks after 6-OHDA is evident in the pioglitazone-treated monkey, compared to placebo. Note that each animal has a unique color scale (units: DV). **c** Treatment group MHED uptake (mean ± SE) in the cardiac left ventricle in three select levels from base to apex and across regions. Treatment × Region × Level ANOVA analysis revealed a significant difference at 12 weeks after neurointoxication (*F*(35, 280) = 2.10, *p* < 0.034, *ηp*^*2*^ = 0.21) and no significant difference at baseline (*F*(35, 280) = 0.70, *p* > 0.711, *ηp*^*2*^ = 0.081) or 1 week (*F*(35, 280) = 0.78, *p* > 0.510, *ηp*^2^ = 0.089). **d** 3D surface plots of group mean MHED uptake in the cardiac left ventricle at baseline, 1, and 12 weeks after 6-OHDA in the two groups across left ventricle regions and levels. The *x*-axis represents eight regions starting with septal and progressing though anterior, lateral, and inferior. The *y*-axis represents radioligand uptake in six levels of the left ventricle descending from base to apex. *z*-axis plot height and corresponding color scale represent values of MHED uptake (DV) at each anatomical area. Polynomial trend analysis did not reach a significant difference based on treatment [Treatment × Region (quadratic) × Level (linear): 33.24%, *p* > 0.084, *ηp*^*2*^ = 0.327]. Simple interaction tests evaluating plot shape independently for each treatment confirmed distinct trends in the Region × Level shape (pioglitazone, quadratic × linear: 67.92%, *p* < 0.019; placebo, cubic × linear: 26.61%, *p* < 0.004).

Adult rhesus macaques were evaluated at baseline, intoxicated with 6-OHDA (50 mg/kg i.v.), and 24 h later randomly assigned to receive daily oral doses of placebo (*n* = 5) or pioglitazone (*n* = 5; 5 mg/kg) (Fig. 1). Feces and weight monitoring, neurological evaluations with a clinical rating scale, activity recording, troponin values, and electrocardiography indicated that 6-OHDA and pioglitazone were well tolerated (Supplementary Figure 1; Supplementary Tables 1, 2, and 3). Echocardiogram analysis revealed small and variable changes in animals in both groups (Supplementary Table 4). At 12 weeks, % fractional shortening was significantly higher in the pioglitazone group (*p* < 0.0001), perhaps because of a higher heart rate (Supplementary Figure 2), although no significant correlation was found (Pearson’s *r* = 0.449, *p* = 0.19).

### MHED uptake by sympathetic terminals varied across cardiac anatomy, over time, and with treatment

Cardiac sympathetic MHED PET data acquired at baseline, 1, and 12 weeks after 6-OHDA were analyzed in eight circumferential regions (septal through inferior) at six axial levels (base to apex) of the left ventricle. Distribution volume maps of the cardiac left ventricle revealed robust MHED uptake at baseline, near complete loss at 1 week after 6-OHDA, and partial recovery by 12 weeks (Fig. 1b). Interestingly, group average MHED uptake values at 12 weeks suggested greater recovery of uptake in pioglitazone animals, dependent on cardiac region and level (Fig. 1c).

To statistically characterize MHED uptake across left ventricle anatomy and between treatment groups, we used repeated measures ANOVA with polynomial trend analysis. 3D surface plots were created to visualize trends for each treatment group at every time point (Fig. 1d). Analysis included calculating percent of variance in each region, level, or combined region and level trend component (linear, quadratic, cubic, and order 4) in all animals. At time points for which ANOVA indicated an effect of pioglitazone, we performed simple interaction tests to characterize polynomial trends for placebo- and pioglitazone-treated groups independently (Table 1).

**Table 1:** MHED PET statistical analysis

MHED PET statistical test	Effect evaluated	Baseline	1 week after 6-OHDA	12 weeks after 6-OHDA
ANOVA (*n* = 10)	Region	*F*(7,56) = 5.21, *p* < 0.006, *η*_p_^2^ = 0.40	NR	*F*(7,56) = 9.31, *p* < 0.001, *η*_p_^2^ = 0.54
	Level	*F*(5,40) = 40.40, *p* < 0.001, *η*_p_^2^ = 0.84	NR	*F*(5,40) = 145.77, *p* < 0.001, *η*_p_^2^ = 0.95
	Region × Level	*F*(35, 280) = 4.48, *p* < 0.001, *η*_p_^2^ = 0.36	*F*(35, 280) = 5.94, *p* < 0.005, *η*_p_^2^ = 0.43	*F*(35,280) = 4.32, *p* < 0.001, *η*_p_^2^ = 0.35
	Treatment × Region × Level	NR	NR	*F*(35,280) = 2.10, *p* < 0.034, *η*_p_^2^ = 0.21
Combined groups polynomial trend analysis (*n* = 10)	Region	Linear: 71.4%, *F*(1,8) = 14.04, *p* < 0.006, *η*_p_^2^ = 0.64	Linear: 56.7%, *F*(1,8) = 4.54, *p* > 0.066, *η*_p_^2^ = 0.36	Quadratic: 64.1%, *F*(1,8) = 27.17, *p* < 0.001, *η*_p_^2^ = 0.77
		Cubic: 15.8%, *F*(1,8) = 3.38, *p* > 0.103, *η*_p_^2^ = 0.30	Cubic: 19.1%, *F*(1,8) = 6.287, *p* < 0.04, *η*_p_^2^ = 0.44	Cubic: 15.3%, *F*(1,8) = 7.97, *p* < 0.022, *η*_p_^2^ = 0.50
	Level	Linear: 72.5%, *F*(1,8) = 56.146, *p* < 0.001, *η*_p_^2^ = 0.875	Linear: 66.0%, *F*(1,8) = 2.09, *p* > 0.186, *η*_p_^2^ = 0.21	Linear: 99.8%, *F*(1,8) = 259.89, *p* < 0.001, *η*_p_^2^ = 0.97
		Quadratic: 27.4%, *F*(1,8) = 42.16, *p* < 0.001, *η*_p_^2^ = 0.84	Quadratic: 32.5%, *F*(1,8) = 11.29, *p* < 0.01, *η*_p_^2^ = 0.59	
	Treatment × Region	NR	NR	Linear: 52.1%, *F*(1,8) = 1.27, *p* > 0.293, *η*_p_^2^ = 0.14
				Quadratic: 17.6%, *F*(1,8) = 0.48, *p* > 0.507, *η*_p_^2^ = 0.06
	Treatment × Level	NR	NR	Linear: 72.8%, *F*(1,8) = 3.281, *p* > 0.11, *η*_p_^2^ = 0.29
				Quadratic: 19.1%, *F*(1,8) = 1.32, *p* > 0.284, *η*_p_^2^ = 0.14
	Treatment × Region × Level	NR	NR	Linear × Linear: 21.4%, *F*(1,8) = 3.31, *p* > 0.106, *η*_p_^2^ = 0.29
				Quadratic × Linear: 33.2%, *F*(1,8) = 3.88, *p* > 0.084, *η*_p_^2^ = 0.33
Simple interaction test placebo (*n* = 5)	Region	NR	NR	Quadratic: 64.4%, *F*(1,4) = 16.53, *p* < 0.015, *η*_p_^2^ = 0.81
				Cubic: 27.0%, *F*(1,4) = 3.63, *p* > 0.13, *η*_p_^2^ = 0.48
	Level	NR	NR	Linear: 99.8%, *F*(1,4) = 154.26, *p* < 0.001, *η*_p_^2^ = 0.98
	Region × Level	NR	NR	Cubic × Linear: 26.6%, *F*(1,4) = 34.02, *p* < 0.004, *η*_p_^2^ = 0.90
				Linear × Linear: 17.1%, *F*(1,4) = 2.22, *p* < 0.037, *η*_p_^2^ = 0.36
Simple interaction test pioglitazone (*n* = 5)	Region	NR	NR	Quadratic: 59.6%, *F*(1,4) = 12.62, *p* < 0.024, *η*_p_^2^ = 0.76
				Linear: 23.3%, *F*(1,4) = 4.99, *p* > 0.089, *η*_p_^2^ = 0.56
	Level	NR	NR	Linear: 98.6%, *F*(1,4) = 106.90, *p* < 0.001, *η*_p_^2^ = 0.96
	Region × Level	NR	NR	Quadratic × linear: 67.9%, *F*(1,4) = 14.31,*p* < 0.019, *η*_p_^2^ = 0.78

Results of repeated measures ANOVA and polynomial trend analysis at baseline, 1 and, 12 weeks after 6-OHDA. ANOVA and combined group polynomial trend analyses were performed with animals from both treatment groups. Polynomial trend analysis for effects of treatment and simple interaction tests for individual treatment groups are reported if a treatment effect was detected by ANOVA. Repeated measures ANOVA results are included if *p* < 0.05. Polynomial trend results are included if contributing ≥15% to the variance of the shape of the surface plot.

*PET* positron emission tomography, *NR* not reported, *MHED* [11 C]meta-hydroxyephedrine, *6-OHDA* 6-hydroxydopamine, *η*_p_^2^ effect size (partial eta squared)

Baseline sympathetic innervation, as identified by MHED and averaged across all animals, differed across cardiac anatomy. Regionally, a significantly linear decrease was observed from septal to inferior regions (71.41%, *p* < 0.006). From base to apex levels, MHED update decrease was largely linear (72.45%, *p* < 0.001), with a smaller quadratic contribution (27.4%, *p* < 0.001). One week after 6-OHDA, MHED uptake diminished dramatically throughout the entire left ventricle. Surface plots of both groups are predominantly flat, illustrating nearly complete and anatomically uniform loss of MHED uptake (Fig. 1d). At this time (1 week), the only significant effect was region in combination with level (Region × Level *p* < 0.005) (Table 1). Pioglitazone treatment did not affect sympathetic innervation at 1 week (*F*(1,8) = 0.345, *p* > 0.573, *η*_p_^*2*^ = 0.41).

12 weeks after 6-OHDA, partial recovery of MHED uptake was evident in all animals, with variations by cardiac region (*p* < 0.001) and level (*p* < 0.001) (Fig. 1d). Compared to placebo, pioglitazone administration produced a unique pattern of sympathetic innervation (Fig. 1d) in combination with region and level (*p* < 0.034; Table 1), although there was no effect of treatment when the left ventricle was analyzed as a whole (*F*(1,8) = 1.012, *p* > 0.344, *η*_p_^2^ = 0.112). Simple interaction tests analyzing region and level effects for each treatment group independently revealed distinct trends in the distribution of sympathetic innervation. In the pioglitazone group, the region by level interaction was almost entirely quadratic by linear [Region (quadratic) × Level (linear): 67.92%, *p* < 0.019] (Table 1; Supplementary Figure 3a), producing a quadratic shape across regions and a linear decrease from basal to apical levels (Fig. 1d). In the placebo group, loss of MHED uptake in the basal lateral region was reflected in the cubic contribution to the region plot [Region (cubic) × Level (linear): 26.61%, *p* < 0.004] (Table 1; Supplementary Figure 3b), seen as decreased surface plot elevation in the basal anterior and lateral regions (Fig. 1d). Decreased uptake in the placebo group was also evident in the apical septal region, producing a flatter plot across apical regions and slightly steeper slope across levels (slope: −0.41), compared to the pioglitazone plot (slope: −0.34).

### PBR28 inflammatory cell uptake was significantly increased at 1 week after systemic 6-OHDA in placebo-treated compared to pioglitazone-treated animals

**Fig. 2:**
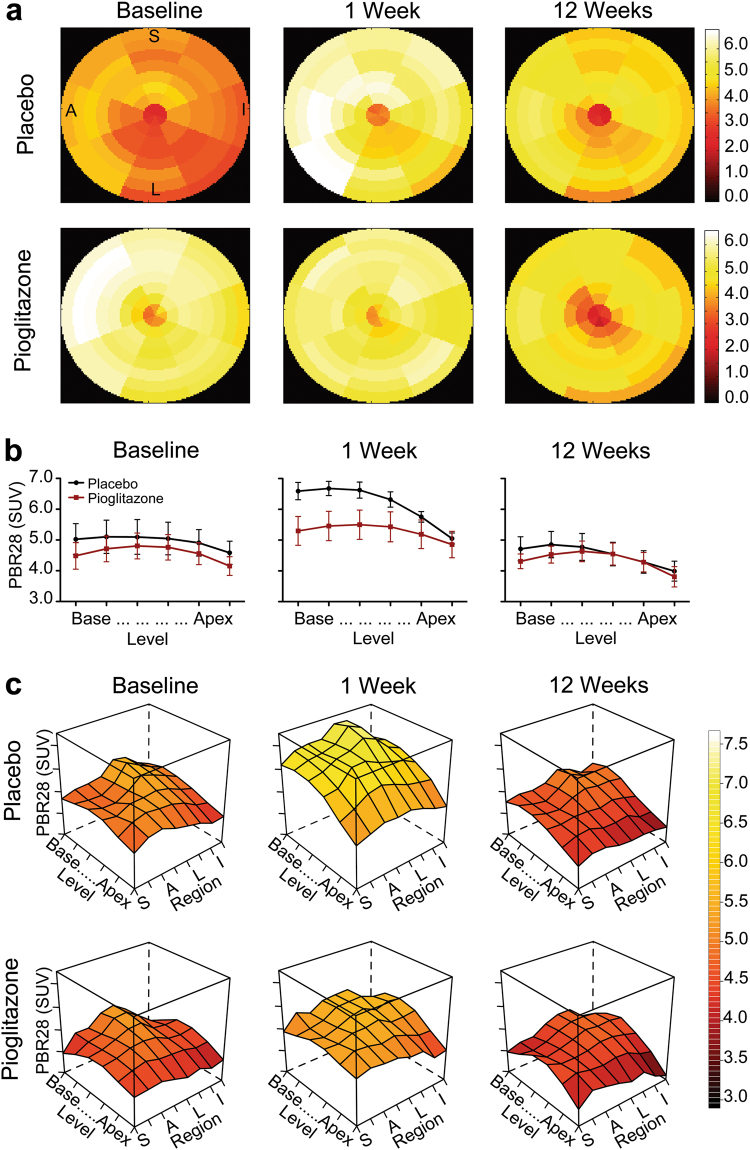
[^11^C]PBR28 PET. **a** Cardiac standard uptake value (SUV) maps of PBR28 uptake at baseline, 1, and 12 weeks after 6-OHDA in representative placebo- and pioglitazone-treated monkeys (apex of heart at center, base of left ventricle at edge). Letters indicate region orientation (S septal, I inferior, L lateral, A anterior). Greater increase in PBR28 uptake from baseline to 1 week after 6-OHDA is visible in the placebo-treated monkey compared to pioglitazone. Note that each animal has a unique color scale (units: SUV). **b** Treatment group PBR28 uptake (mean ± SE) from base to apex, with each left ventricle level averaged over regions. A treatment effect in combination with level (Treatment × Level ANOVA: *F*(5,40) = 6.44, *p* < 0.002, *η*_p_^2^ = 0.45) was present at 1 week, which was not significant at baseline (*F*(5,40) = 0.54, *p* > 0.662, *η*_p_^2^ = 0.063) or 12 weeks (*F*(5,40) = 1.40, *p* > 0.265, *η*_p_^2^ = 0.149). PBR28 uptake significantly differed over time (*F*(2,16) = 12.9, *p* < 0.001, *η*_p_^2^ = 0.604). Considering the left ventricle as a whole, placebo-treated animals exhibited a significant increase in PBR28 uptake from baseline (4.96 ± 0.44) to 1 week (6.17 ± 0.18; *t*(8) = −3.069, *p* < 0.046, *d*_*z*_ = 1.37). In contrast, uptake was similar between baseline (4.58 ± 0.34) and 1 week (5.28 ± 0.41) in pioglitazone-treated animals (*t*(8) = 1.802, *p* < 0.33, *d*_*z*_ = 0.8). By 12 weeks (placebo: 4.52 ± 0.34; pioglitazone: 4.35 ± 0.27) neither group showed a significant difference from baseline. **c** 3D surface plots of group mean PBR28 uptake at baseline, 1, and 12 weeks after 6-OHDA in placebo- and pioglitazone-treated animals across left ventricle regions and levels. The *x*-axis represents eight regions starting with septal and progressing through anterior, lateral, and inferior. The *y*-axis represents radioligand uptake in six levels of the left ventricle descending from base to apex. *z*-axis plot height and corresponding color scale represent values of PBR28 uptake (SUV) at each anatomical area. Polynomial trend analysis revealed a significant difference based on treatment [Treatment × Level (linear): 92.46%, *p* < 0.013] at 1 week. Simple interaction tests evaluating uptake plot shape independently for each treatment confirmed distinct trends in the distribution of PBR28 uptake by level related to treatment (pioglitazone, quadratic: 52.87%, *p* < 0.029; placebo, linear: 79.46%, *p* < 0.005).

PET scans with PBR28 to assess inflammatory cell infiltration in the left ventricle were obtained at baseline, 1, and 12 weeks after 6-OHDA and analyzed using the same statistical methods as MHED. Standard uptake value maps of the cardiac left ventricle revealed some PBR28 uptake in all animals at baseline, followed by an increase at 1 week after neurointoxication, which was noticeably higher in the placebo group (Fig. 2a). At 12 weeks after 6-OHDA, PBR28 uptake had returned to near baseline for both groups. Interestingly, at 1 week, group average PBR28 uptake values suggested different anatomical patterns of inflammation between groups (Fig. 2b). We investigated this pattern using ANOVA with polynomial trend analysis and surface plots (Table 2, Fig. 2c).

**Table 2:** PBR28 PET statistical analysis

PBR28 PET statistical test	Effect evaluated	Baseline	1 week after 6-OHDA	12 weeks after 6-OHDA
ANOVA (*n* = 10)	Region	*F*(7,56) = 7.17, *p* < 0.001, *η*_p_^2^ = 0.47	*F*(7,56) = 5.34, *p* < 0.002, *η*_p_^2^ = 0.40	*F*(7,56) = 2.85, *p* < 0.017, *η*_p_^2^ = 0.26
	Level	*F*(5,40) = 10.93, *p* < 0.001, *η*_p_^2^ = 0.58	*F*(5,40) = 27.27, *p* < 0.001, *η*_p_^2^ = 0.77	*F*(5,40) = 19.39, *p* < 0.001, *η*_p_^2^ = 0.71
	Region × Level	*F*(35,280) = 4.94, *p* < 0.001, *η*_p_^2^ = 0.38	*F*(35,280) = 2.36, *p* < 0.032, *η*_p_^2^ = 0.23	*F*(35,280) = 5.57, *p* < 0.001, *η*_p_^2^ = 0.41
	Treatment × Level	NR	*F*(5, 40) = 6.44, *p* < 0.002, *η*_p_^2^ = 0.45	NR
	Treatment × Region × Level	*F*(35,280) = 2.21, *p* < 0.015, *η*_p_^2^ = 0.22	NR	NR
Combined groups polynomial trend analysis (*n* = 10)	Region	Quadratic: 81.1%, *F*(1,8) = 14.14, *p* < 0.006, *η*_p_^2^ = 0.64	Quadratic: 89.0%, *F*(1,8) = 13.04, *p* < 0.007, *η*_p_^2^ = 0.62	Quadratic: 62.4%, *F*(1,8) = 6.77, *p* < 0.032, *η*_p_^2^ = 0.46
				Cubic: 20.1%, *F*(1,8) = 4.27, *p* > 0.073, *η*_p_^2^ = 0.35
	Level	Quadratic: 60.6%, *F*(1,8) = 20.05, *p* < 0.002, *η*_p_^2^ = 0.72	Linear: 71.5%, *F*(1,8) = 32.83, *p* < 0.001, *η*_p_^2^ = 0.80	Linear: 60.4%, *F*(1,8) = 19.87, *p* < 0.002, *η*_p_^2^ = 0.71
		Linear: 38.7%, *F*(1,8) = 6.98, *p* < 0.030, *η*_p_^2^ = 0.47	Quadratic: 28.5%, *F*(1,8) = 22.35, *p* < 0.001, *η*_p_^2^ = 0.74	Quadratic: 39.4%, *F*(1,8) = 23.75, *p* < 0.001, *η*_p_^2^ = 0.75
	Treatment × Region	Cubic: 60.6%, *F*(1,8) = 3.03, *p* > 0.12, *η*_p_^2^ = 0.28	Quadratic: 87.1%, *F*(1,8) = 1.626, *p* > 0.24, *η*_p_^2^ = 0.17	NR
		Linear: 18.4%, *F*(1,8) = 0.50, *p* > 0.5, *η*_p_^2^ = 0.06		
	Treatment × Level	Quadratic: 84.3%, *F*(1,8) = 1.36, *p* > 0.28, *η*_p_^2^ = 0.15	Linear: 92.5%, *F*(1,8) = 10.04, *p* < 0.013, *η*_p_^2^ = 0.56	NR
	Treatment × Region × Level	Quadratic × Linear: 42.0%, *F*(1,8) = 4.78, *p* > 0.06, *η*_p_^2^ = 0.37	Linear × Linear: 21.7%, *F*(1,8) = 1.23, *p* > 0.299, *η*_p_^2^ = 0.13	NR
		Linear × Linear: 24.2%, *F*(1,8) = 7.37, *p* < 0.26, *η*_p_^2^ = 0.48		
Simple interaction test placebo (*n* = 5)	Region	Quadratic: 75.3%, *F*(1,4) = 3.27, *p* > 0.145, *η*_p_^2^ = 0.45	Quadratic: 91.7%, *F*(1,4) = 7.05, *p* < 0.057, *η*_p_^2^ = 0.64	NR
	Level	Linear: 59.2%, *F*(1,4) = 4.6, *p* > 0.099, *η*_p_^2^ = 0.53	Linear: 79.5%, *F*(1,4) = 33.10, *p* < 0.005, *η*_p_^2^ = 0.89	NR
		Quadratic: 39.4%, *F*(1,4) = 4.99, *p* > 0.089, *η*_p_^2^ = 0.56	Quadratic: 20.5%, *F*(1,4) = 12.21, *p* < 0.025, *η*_p_^2^ = 0.75	
	Region × Level	Linear × Linear: 42.3%, *F*(1,4) = 65.10, *p* < 0.001, *η*_p_^2^ = 0.94	Cubic × Linear: 18.2%, *F*(1,4) = 0.95, *p* > 0.386, *η*_p_^2^ = 0.19	NR
		Cubic × Linear: 28.5%, *F*(1,4) = 5.27, *p* > 0.083, *η*_p_^2^ = 0.57		
Simple interaction test pioglitazone (*n* = 5)	Region	Quadratic: 74.7%, *F*(1,4) = 59.33, *p* < 0.002, *η*_p_^2^ = 0.94	Quadratic: 76.7%, *F*(1,4) = 8.859, *p* < 0.040, *η*_p_^2^ = 0.69	NR
	Level	Quadratic: 76.6%, *F*(1,4) = 17.65,*p* < 0.014, *η*_p_^2^ = 0.82	Quadratic: 52.9%, *F*(1,4) = 11.01, *p* < 0.029, *η*_p_^2^ = 0.73	NR
		Linear: 23.1%, *F*(1,4) = 2.56, *p* > 0.185, *η*_p_^2^ = 0.39	Linear: 47.0%, *F*(1,4) = 4.08, *p* > 0.113, *η*_p_^2^ = 0.51	
	Region × Level	Quadratic × Linear: 63.5%, *F*(1,4) = 8.72, *p* < 0.042, *η*_p_^2^ = 0.69	linear × Linear: 33.1%, *F*(1,4) = 7.12, *p* > 0.056, *η*_p_^2^ = 0.64	NR

Results of repeated measures ANOVA and polynomial trend analysis at baseline, 1, and 12 weeks after 6-OHDA. ANOVA and combined group polynomial trend analyses were performed with animals from both treatment groups. Polynomial trend analysis for effects of treatment and simple interaction tests for individual treatment groups are reported if a treatment effect was detected by ANOVA. Repeated measures ANOVA results are included if *p* < 0.05. Polynomial trend results are included if contributing ≥15% to the variance of the shape of the surface plot.

*PET* positron emission tomography, *NR* not reported, *PBR28* *N*-(2-[11 C]methoxybenzyl)-*N*-(4-phenoxypyridin-3-yl)acetamide, *6-OHDA* 6-hydroxydopamine, *η*_p_^2^ effect size (partial eta squared)

At baseline, PBR28 uptake over the entire left ventricle was similar between groups (*F*(1,8) = 0.381, *p* > 0.554, *η*_p_^*2*^ = 0.045), peaking in the basal lateral area (Fig. 2c). Uptake was dependent upon the circumferential region (*p* < 0.001), base to apex level (*p* < 0.001), and combined region and level (*p* < 0.001) (Table 2). Interestingly, a difference in anatomical PBR28 uptake distribution related to treatment group was observed at baseline (Treatment × Region × Level *p* < 0.015) (Table 2). Follow-up polynomial trend analyses revealed that the groups differed only in the Region (linear) by Level (linear) component (*p* < 0.026; Table 2).

One week after 6-OHDA, similar to baseline, the effects of region (*p* < 0.002) and level (*p* < 0.001), as well as an interaction of region with level (*p* < 0.032) remained significant. Notably, PBR28 uptake was higher in placebo-treated animals compared to pioglitazone in a manner dependent on the anatomical level (*p* < 0.002) (Fig. 2, Table 2). Simple interaction tests using trend analysis for each treatment group revealed distinct trends across levels. For the pioglitazone group, the plot was flattened from base to apex, with the level portion mostly quadratic (52.87%, *p* < 0.029) in shape. In contrast, in the placebo group the dramatically higher PBR28 uptake in the left ventricle base produced a more linear level plot (79.46%, *p* < 0.005) (Fig. 2c, Table 2). This effect can also be seen in the steeper slope across levels of the placebo group plot (slope: −0.31) compared to the pioglitazone group plot (slope: −0.09).

To investigate whether the PBR28 Treatment × Level effect at 1 week was caused by group difference at baseline, we evaluated percent increase of PBR28 uptake from baseline to 1 week. We found that percent increase was significantly higher in the placebo group across levels of the heart (*p* < 0.008) and for region combined with level (*p* < 0.001) (Supplementary Figure 4). We also compared PBR28 uptake at baseline to 1 week and found a significant increase in placebo- (*p* < 0.046), but not pioglitazone-treated (*p* < 0.33) animals (Fig. 2).

At 12 weeks after 6-OHDA, PBR28 uptake returned to near baseline values for both groups, with anatomical distribution similar to baseline (Fig. 2, Table 2). No significant effect of treatment was observed at 12 weeks after neurointoxication (Fig. 2c).

### ATSM measures of cardiac oxidative stress changed over time and were modulated by PPARγ activation

**Fig. 3:**
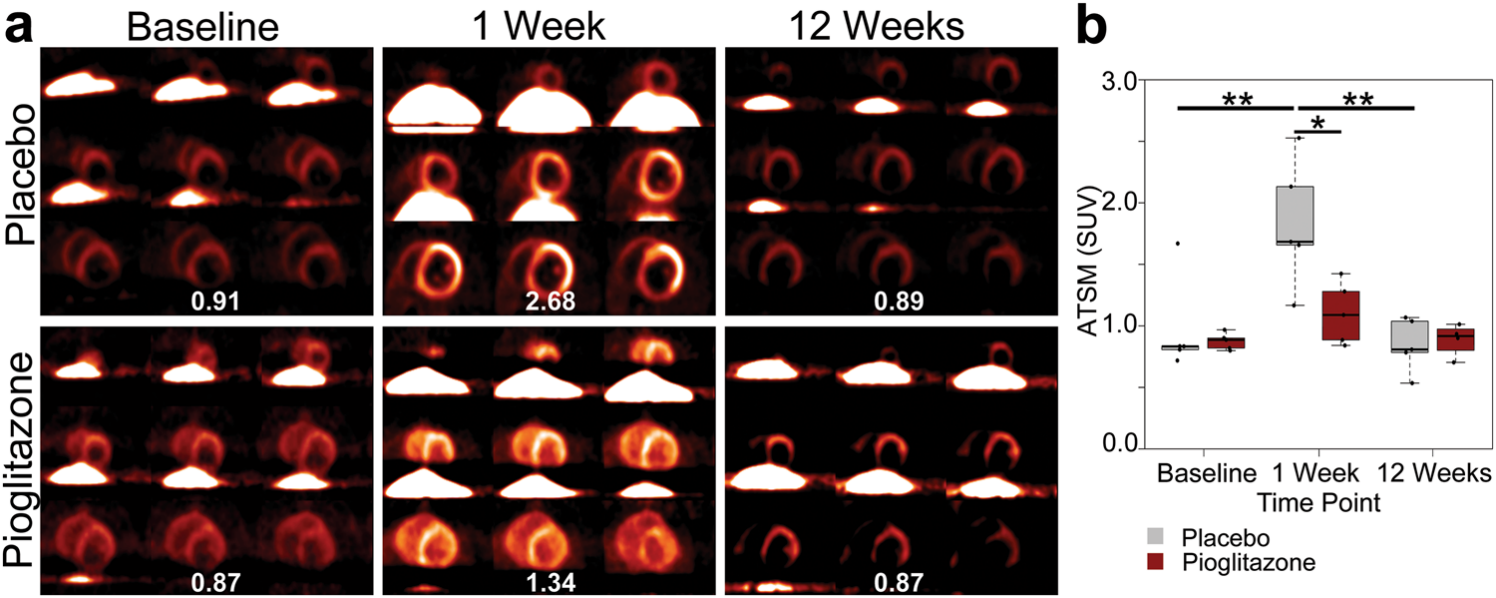
[^61^Cu]ATSM PET. **a** ATSM radioligand uptake images at baseline, 1, and 12 weeks after 6-OHDA in representative placebo- and pioglitazone-treated monkeys. A greater increase of ATSM uptake from baseline to 1 week was seen in placebo-treated animals compared to pioglitazone. Note that the color scale is unique to each animal; ATSM numerical data included for clarification. Data were reported only for the anterior region due to high uptake in the liver, visible in the ATSM images. **b** Box-and-whisker plot of ATSM uptake at baseline, 1, and 12 weeks after 6-OHDA. Whiskers extend to the most extreme data point that is no more than 1.5× the interquartile range. The increase in ATSM uptake from baseline (placebo median: 0.829; pioglitazone median: 0.822) to 1 week (placebo median: 1.681; pioglitazone median: 1.085) was significant in placebo- (**Z = 2.02, *p* < 0.044, *r* = 0.903), but not pioglitazone-treated animals (*Z* = 1.214, *p* > 0.225, *r* = 0.543). By 12 weeks, neither group showed a significant difference from baseline (placebo median: 0.804; pioglitazone median: 0.914). The decrease from 1 week to 12 weeks was significant for placebo-treated animals (**Z = 2.02, *p* < 0.044, *r* = 0.903), but not for the pioglitazone group (*Z* = 0.730, *p* > 0.465, *r* = 0.233). Note the statistically significantly greater ATSM uptake in placebo- compared to pioglitazone-treated monkeys at 1 week after 6-OHDA (*, *U* = 2, *p* < 0.033, *r* = 0.693). No significant difference between treatment groups was found at baseline (*U* = 10, *p* > 0.690, *r* = 0.165) or 12 weeks (*U* = 10, *p* = 1, *r* = 0).

Cardiac oxidative stress was evaluated by ATSM PET at baseline, 1, and 12 weeks after 6-OHDA. ATSM uptake was quantified in all animals from base to apex in the anterior region of the heart because data acquisition in other regions was obscured by liver uptake. At baseline, ATSM uptake was similar between the placebo (median = 0.829) and pioglitazone (median = 0.882) groups (Mann–Whitney *U* = 10, *p* = 0.690, *r* = 0.165) (Fig. 3). From baseline to 1 week, ATSM uptake increased significantly in placebo- (*p* < 0.044), but not pioglitazone-treated animals (*p* > 0.225) (Fig. 3). This produced a significant difference between groups at 1 week (placebo median = 1.681; pioglitazone median = 1.085; *U* = 2, *p* = 0.032, *r* = 0.693) (Fig. 3). At 12 weeks, ATSM uptake did not differ from baseline levels in either placebo (median = 0.804) or pioglitazone (median = 0.914) groups; no difference in uptake between groups was detected (*U* = 10, *p* = 1, *r* = 0) (Fig. 3).

### In vivo MHED PET data correlate with post mortem evaluation of sympathetic innervation

**Fig. 4:**
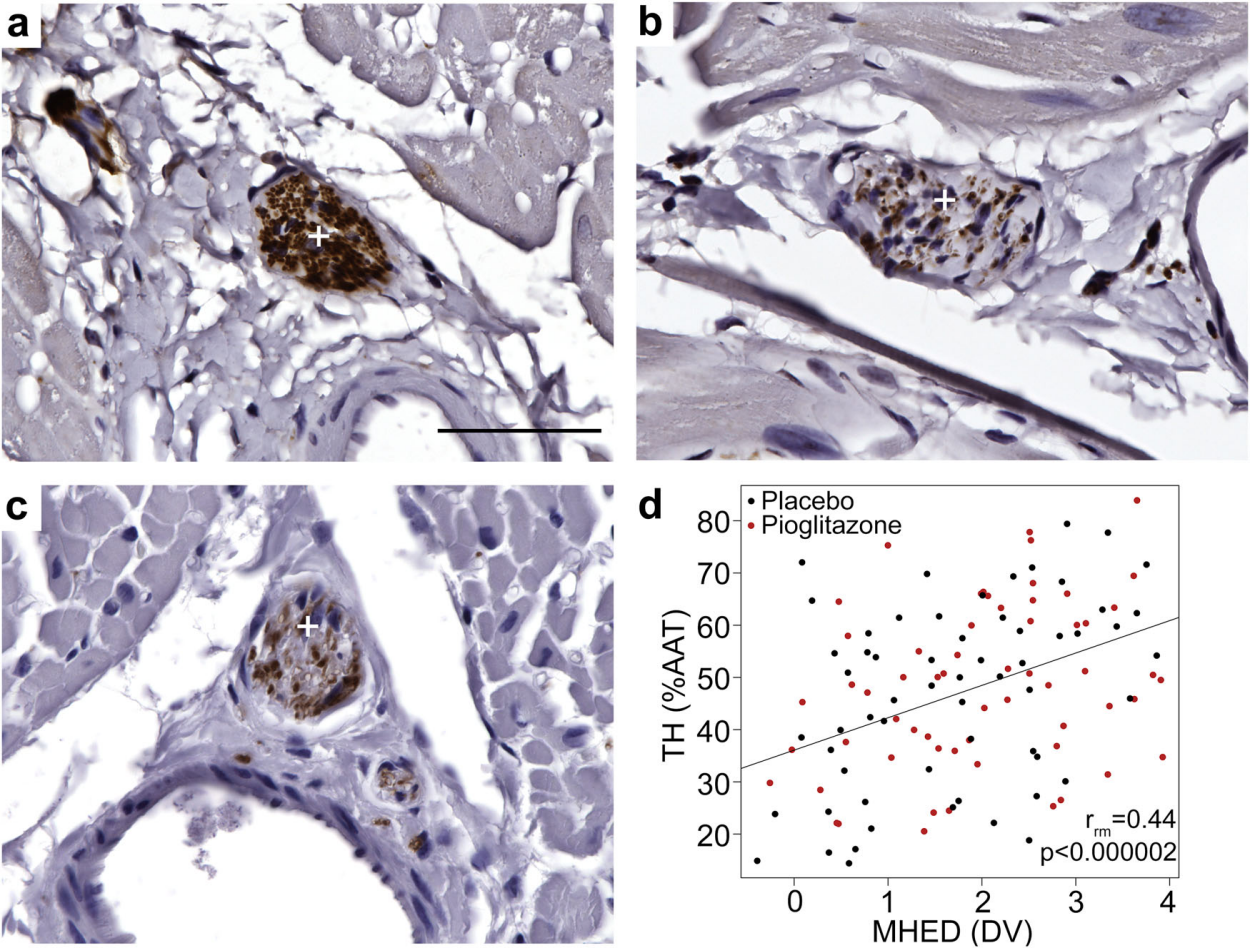
Post mortem evaluation of cardiac sympathetic innervation. **a**–**c** Photomicrographs of cardiac left ventricle sections immunostained with the sympathetic innervation marker tyrosine hydroxylase (TH) and counterstained with hematoxylin. **a** Corresponds to controls, **b** placebo-, and (**c**) pioglitazone-treated monkeys. TH-immunoreactivity (−ir) was observed in bundles of nerves (white+), typically adjacent to blood vessels, and in individual nerve fibers, as previously described (Joers et al., 2014). Qualitatively, TH-ir was robustly present in nerve bundles and fibers of healthy controls and diminished in 6-OHDA-treated subjects. **d** Plot of repeated measures correlation of cardiac nerve bundle TH-ir percent area above threshold (%AAT) with 12-week MHED uptake in placebo- and pioglitazone-treated animals. Each point represents a single anatomical area in one animal (12 total anatomical areas (4 regions × 3 levels) per animal; 10 animals). A significant repeated measures correlation was found between TH-ir and 12-week MHED uptake in all ten animals (*r*_rm_ = 0.44, *p* < 0.000002). This correlation was also significant when treatment groups were analyzed separately and was similar between groups (placebo: *r*_rm_ = 0.47, *p* < 0.0004; pioglitazone: *r*_rm_ = 0.41, *p* < 0.002). 12-week MHED PET data included in the repeated measures correlation were matched to levels and regions analyzed by post mortem immunohistochemistry (MHED PET levels 1, 4 and 6; MHED PET regions SA, AL, LI and IS). Scale bar = 50 μm.

Hearts from the ten 6-OHDA-treated animals were collected at least 72 h after final PET scan and compared to five normal controls. Sections at the base, middle, and apex levels of each left ventricle were evaluated in the septal, anterior, lateral, inferior regions. Antibodies against tyrosine hydroxylase (TH) and PGP9.5 were used to identify sympathetic innervation and validate neurodegeneration, respectively (Fig. 4; Supplementary Figure 5; Supplementary Table 5). As PET imaging showed that PBR28 and ATSM returned to baseline values by 12 weeks (Figs. 2 and 3), post mortem data on inflammation and oxidative stress are not included. A significant correlation across cardiac levels and regions was observed between TH-immunoreactivity in cardiac nerve bundles (percent area above threshold, %AAT) and 12-week MHED uptake (*r*_rm_ = 0.44, *p* < 0.000002; Fig. 4d).

## Discussion

The present study in rhesus macaques demonstrated that PET with the radioligands MHED, PBR28, and ATSM successfully detected changes over time and across the cardiac left ventricle in sympathetic innervation, inflammatory response, and oxidative stress during neurotoxin-induced neurodegeneration and PPARγ-associated neuroprotection.

Loss of cardiac sympathetic innervation is an increasingly recognized feature of PD,^9^ which can be mimicked in nonhuman primates by systemic administration of 6-OHDA.^13,14^ Compared to systemic MPTP (1-methyl-1,2,3,6-tetrahydropiridine^27^), systemic 6-OHDA administration to rhesus macaques induces a reproducible and persistent sympathetic denervation of the heart. Furthermore, 6-OHDA-induced mechanisms of neurodegeneration^15^ resemble PD-associated recruitment of inflammatory cells and increased production of ROS,^11,12^ making the rhesus systemic 6-OHDA model a suitable platform for evaluation of imaging biomarkers during cardiac sympathetic loss. The high spatial resolution of the microPET scanner (2 mm)^28^ enabled extraction of 48 anatomical areas (8 regions × 6 levels), which combined with standard polynomial trend analysis provided in-depth characterization of radioligand distribution across the rhesus cardiac left ventricle. In the clinical setting, metaiodobenzylguanidine (MIBG) scintigraphy has been preferred as a cardiac sympathetic imaging technique due to its relatively easy availability and low cost.^9^ However, scintigraphy has reduced detection sensitivity and poor spatial resolution compared to single-photon emission computed tomography (15 mm) or PET (5 mm),^29^ limiting its application for detailed 3D mapping of radioligand uptake.

In normal, healthy human subjects, cardiac sympathetic innervation is decreased in the inferior region relative to the anterior, and in the apex relative to the base, as documented by multiple methods including MHED PET,^30^ MIBG,^31^ and histology.^32^ In the present study using nonhuman primates, baseline MHED distribution mirrored naturally occurring anatomical heterogeneity in the left ventricular myocardium of humans, with lowest uptake in the inferior region and apical level. A previous MHED evaluation of cardiac sympathetic innervation in normal rhesus did not reveal statistically significant differences between myocardial regions,^14^ probably because standard PET applied to the study lacks the higher resolution of microPET. Interestingly, follow-up post mortem evaluation of control monkeys identified differences in regional sympathetic nerve fiber density, with inferior and lateral walls exhibiting the least TH-ir fiber density;^13^ this suggests that the heterogeneous pattern of cardiac innervation is shared across human and nonhuman primate species. In our control animals, analysis of nerve bundles did not detect regional differences (Supplementary Figure 5). Future work quantifying sympathetic nerve fiber density, in addition to markers of inflammation and oxidative stress, is warranted but beyond the scope of this publication.

Cardiac sympathetic neurodegeneration in PD is anatomically heterogeneous. The apex appears more affected than the base,^2,33^ with sparing of the anterior and proximal regions.^2,34^ Over time, the loss becomes more diffuse, increasingly affecting the anterior and septal regions.^3^ As in PD, placebo-treated monkeys at 12 weeks exhibited greater loss of sympathetic innervation in the apex relative to the base and greatest regional loss in the inferior wall, as detected by both MHED PET (Fig. 1) and TH immunohistochemistry (Supplementary Figure 5). This heterogeneous pattern is in accord with a prior report from our research group,^14^ demonstrating both replicability of the model and cardiac vulnerability for sympathetic loss at specific levels and regions.

MHED PET demonstrated a replicable time course of 6-OHDA-induced cardiac sympathetic lesion across animals and previous investigations,^14^ with minimal uptake at 1 week after neurointoxication and some recovery by 12 weeks. Following the predicted timing of neurodegeneration, PET imaging captured ongoing changes in inflammation and oxidative stress elicited by the neurotoxin and attenuated by the PPARγ agonist. Reproducibility of the rhesus 6-OHDA model, both in time course and in sympathetic effects, further highlights its utility as a study platform for biomarkers of cardiac neurodegeneration and neuroprotection.

PBR28 imaging detected anatomically heterogeneous inflammation in the heart associated with cardiac sympathetic neurodegeneration. The advantage of using PBR28 to visualize inflammation over TSPO first-generation ligands, such as PK11195, is its increased signal-to-noise ratio due to greater specific binding.^35^ One potential limitation of TSPO-binding radioligands is a nucleotide polymorphism in the TSPO gene (rs6971).^36,37^ This codominant monogenic trait leads to a 2:1 difference in PBR28 PET signal between high and middle affinity binders, making genotyping important for clinical applications. This polymorphism has not been reported in nonhuman primates, although high PBR28 binding variability in baboons has been described.^38^ As PBR28 baseline data in our study did not show outliers, we did not perform genotyping. Consistent with previous reports,^16,39^ naturally occurring PBR28 cardiac uptake was observed at baseline. Anatomically, it was greater at the base than at the apex and varied across regions, which may be related to cardiac physiology and anatomy (Fig. 2). The action of the beating heart drains the leukocyte-laden cardiac lymphatic system from apex to base,^40^ potentially excluding inflammatory cells from apical levels. In addition, right and left coronary arteries originate at the aortic sinuses of the aortic root, prior to branching and decreasing in diameter as they descend toward the cardiac apex. The larger size of the coronary arteries at the base might allow for a larger number of TSPO-expressing circulating cells. These anatomical effects may have also contributed to the elevated PBR28 uptake at the base of the heart in the placebo group at 1 week after 6-OHDA.

Imaging oxidative stress in vivo has been elusive. ATSM is a promising PET agent for oxidative stress because it accumulates in electron-rich areas after reduction of the radioactive copper in the radioligand.^22,23^ In the present study, ATSM uptake in the placebo group paralleled PBR28 imaging of inflammation, with a dramatic increase at 1 week after 6-OHDA and a return to baseline levels for all animals by 12 weeks (Fig. 3). 6-OHDA increases oxidative stress by intraneuronal autoxidation and impairing mitochondrial activity. Our present results thus demonstrate that ATSM is a sensitive radioligand for the detection of increased cardiac oxidative stress after 6-OHDA-induced loss of postganglionic sympathetic innervation to the heart.

The loss of MHED uptake detected at 12 weeks was attenuated in the anterior-lateral region of the base and the septal region of the apex of pioglitazone-treated animals compared to placebo, which correlated with post mortem TH immunohistochemistry (Fig. 4). The same animals presented less PBR28 and ATSM uptake at 1 week, suggesting that the sympathetic neuroprotective effect of pioglitazone was related to modulation of the inflammatory response and accumulation of reactive oxygen species. Pioglitazone is an agonist of the type II nuclear transcription factor PPARγ. By directly inhibiting the transcription factor NFκB, PPARγ plays a key role in the development of the M2 (alternatively activated) anti-inflammatory activation state of monocytes and macrophages, as opposed to the M1 (classically activated) proinflammatory phenotype,^41,42^ promoting neuroprotection. It should be noted that the M1/M2 designation simplifies what is more accurately described as a spectrum of activation state phenotypes determined by immune cell protein expression.^43^ Notably, TSPO radioligands do not distinguish between M1 and M2 cells.^44^ The significant difference in PBR28 uptake at 1 week after 6-OHDA in the cardiac base induced by pioglitazone administration thus suggests that the drug treatment affected immune cell influx, in addition to potential anti-inflammatory M2 programming. PPARγ activation also reduces oxidative stress via increasing expression of the enzymatic antioxidants catalase and superoxide dismutase (SOD) through binding to promoter region PPARγ response elements.^26^ The pioglitazone-induced attenuation of oxidative stress in the heart, detected as decreased ATSM uptake 1 week after neurointoxication, may have further contributed decreased inflammatory cell recruitment, by reducing neuronal damage.

Pioglitazone has been shown to be neuroprotective in rodent models of nigral dopaminergic neurodegeneration induced by systemic MPTP.^45^ In rhesus macaques, pioglitazone (5 mg/kg PO, the same dose used in the present study) has been shown to ameliorate nigral dopaminergic loss induced by unilateral intracarotid artery delivery of MPTP;^46^ preservation of nigral dopaminergic neurons in the pioglitazone animals correlated with a decreased number of nitrotyrosine-positive cells (a marker of oxidative stress) and was associated with decreased microglial activation compared to placebo. As in the present study, pioglitazone dosing was initiated 24 h after neurotoxin challenge, aiming to mimic early but already ongoing catecholaminergic neurodegeneration. In PD patients, pioglitazone failed to prevent progression of motor symptoms.^47^ However, a retrospective study showed that patients with type II diabetes treated with glitazones had a 28% decreased risk of developing PD compared to patients taking other anti-diabetic medication.^48^ This apparent discrepancy in the neuroprotective efficacy of pioglitazone may be related to the current dependency of PD diagnosis on the presence of typical motor symptoms, which emerge when 30–50% of nigral dopaminergic neurons and 30–70% of striatal nerve terminals have been lost.^49–52^ Identification of patients at risk of developing PD, before the onset of overt movement disorder, may provide a more optimal window of time for effective neuroprotection. In vivo imaging may provide an opportunity to visualize and quantify mechanisms of PD neurodegeneration and target validation of treatments aiming to modulate inflammation and oxidative stress.

Although the present study focused on sympathetic neurodegeneration in the heart in PD, this cardiac pathology has also been recognized as a common feature that seriously affects the daily life of patients with other disorders, including idiopathic REM sleep disorder,^53^ pure autonomic failure,^54^ and diabetes.^55^ Advances in identifying cardiac nerve loss biomarkers and treatments can benefit multiple patient populations.

In conclusion, neurodegeneration and PPARγ-associated neuroprotection of postganglionic sympathetic innervation to the heart can be visualized and quantified in vivo and over time using MHED, PBR28, and ATSM PET imaging. Our findings strongly support future preclinical and clinical studies using these radioligands to evaluate the role of inflammation and oxidative stress in peripheral sympathetic neurodegeneration.

## Methods

### Subjects

The study was performed in accordance with the recommendations of the National Research Council Guide for the Care and Use of Laboratory Animals (2011) in an AAALAC accredited facility (Wisconsin National Primate Research Center). Experimental procedures were approved by the UW–Madison Institutional Animal Care and Use Committee of the University (protocol G00705). Ten adult, male rhesus macaques (*Macaca mulatta*) were used. After baseline measures were acquired (see below), animals received systemic 6-OHDA (50 mg/kg i.v.) as previously described.^14^ For the procedure, monkeys were food-deprived overnight; anesthesia was induced with ketamine HCl (15 mg/kg i.m.) and maintained with 1–3% isoflurane in 100% O_2_ at 1 l/min. After 24 h, animals were randomly assigned to receive daily oral dosing of placebo (*n* = 5; 6.2–13.0 years; 9.8–12.3 kg) or pioglitazone (5 mg/kg; *n* = 5; 5.6–11.4 years; 9.4–10.6 kg). For post mortem analysis, hearts from normal, healthy, age and sex matched control rhesus macaques (*n* = 5; 6.8–12.3 years; 7.0–12.6 kg) were obtained from the WNPRC tissue bank.

One placebo-treated animal died after an episode of bradycardia during MHED PET scan 8 days after 6-OHDA and was replaced to reach *n* = 5 for the placebo group. Clinically, the animal exhibited dehydration and severe azotemia. ECGs performed 6 days after neurointoxication revealed abnormalities including prolonged QRS duration and peaked T waves. Histological evaluation of the heart did not reveal abnormalities; proximal renal tubular degeneration and centrilobular hepatocellular degeneration were observed, suggesting as cause of death renal failure potentially due to adverse reaction to anesthesia. Additionally, one pioglitazone-treated animal was excluded from 12-week ATSM data due to subcutaneous radioligand administration.

### Clinical evaluations

Food intake and feces were monitored daily by trained personnel; characteristics were recorded using a descriptive scale.^14^ Presence of PD signs was monitored by a trained observer blind to treatment group using a previously validated clinical rating scale.^56^ General activity recordings,^46^ blood sampling for plasma troponin I, 10-lead ECG (Hewlett-Packard PageWriter), and echocardiograms (LOGIQe, GE Healthcare) were obtained at baseline, 1, and 12 weeks after neurointoxication under ketamine HCl (15 mg/kg i.m.) and analyzed as previously described.^14^ We calculated corrected QT interval (QTc) using the QT120 formula designed for evaluation of rhesus macaques as QT interval/sqrt (RR interval), where RR interval = 120/h.^57^

### MHED, PBR28, and ATSM production

Radioligands were produced at the UW–Madison Medical Physics cyclotron. Production of [^11^C]MHED was performed as previously described.^14^ [^11^C]PBR28 (*N*-(2-[^11^C]methoxybenzyl)-*N*-(4-phenoxypyridin-3-yl)acetamide) was synthesized according to a previously described procedure,^58^ amended to perform in-house. The chemical precursor was methylated with [^11^C]methyl triflate. Next, product was purified with HPLC, diluted in water, and passed through a sterile filtration unit in preparation for injection. The final formulation was typically in excess of 50 mCi with specific activity >9000 mCi/μmol. Several positron-emitting radioisotopes of Cu are available for radiolabeling ATSM. We selected ^61^Cu for favorable half-life (*t*_1/2_ = 3.4 h) and high positron branching ratio (61%). The ^61^Cu was produced by 8 MeV deuteron irradiation of electroplated ^60^Ni. The Ni was dissolved in concentrated HCl (Fisher Optima); this solution was diluted to 6 N, loaded onto an equilibrated AG1-X8 (Bio-Rad 100–200 mesh) anion exchange column, and rinsed with 25 mL 6 N HCl to collect the Ni. Next, radioactive Cu was eluted in 0.1 N HCl, followed by drying of the volume; the ^61^Cu was taken up in 300 μL 3 N HCl and reacted with 20 μL (2 mg/mL in DMSO) of H2-ATSM (ABX) at room temp for 2 min. This solution was loaded onto a prepared C18 light column SEP PAK (Waters), rinsed with 10 mL of 18 MOhm H_2_O (Millipore), and eluted with 0.5 mL ethanol. Finally, saline was added to make 10% ethanol:90% saline prior to 0.22-μm filtration and injection.^23,59^

### PET imaging

PET with MHED, PBR28, or ATSM was performed at baseline, 1, and 12 weeks after 6-OHDA. At 1 week after neurointoxication, ATSM was performed first, followed by MHED 96 h later, and then PBR28 24 h later. At baseline and 12 weeks the PET order varied; a clearance period of at least 24 h (MHED and PBR28) or 96 h (ATSM) was allowed before the next scan. Monkeys underwent PET under isoflurane anesthesia (1–3% in 100% O_2_, 1 L/min); vital signs (respiration, temperature, heart rate) were monitored throughout as previously described.^14^ Animals were positioned supine in a Siemens Focus 220 microPET scanner, using a custom-made bed extension and foam positioning apparatus. After a 15-min transmission scan, radioligand was injected as an i.v. bolus (~5.2 mCi) over 30 s. Dynamic PET images were obtained for 1 h with conventionally increasing frame durations (6 × 30 s, 3 × 60 s, 2 × 120 s, 10 × 300 s).

### PET data analysis

For MHED analysis, whole-blood tracer concentrations were obtained from a volume of interest in the upper left ventricle chamber. Radioligand uptake was quantified using equilibrium distribution volume (DV) of left ventricle tissue relative to whole blood as previously described.^14^ A DV value of 1 corresponds to no excess capacity in tissue relative to blood; DV − 1 thus provides a measure of density of nerve terminals that give the tissue its excess capacity. DV was evaluated in eight sectors (regions) in each of eight short-axis rings (levels) evenly spaced along the long axis of the heart. Data used for statistical analysis included all eight regions from levels 2 to 7 (total: 48 anatomical areas) at each time point. PBR28 and ATSM were evaluated using standard uptake values (SUVs), calculated as the ratio of tissue radioactivity concentration at a given time point and injected activity divided by body weight. For PBR28, SUVs were calculated in the same 48 myocardial areas and quantified using data from the first postinjection hour. ATSM SUVs were calculated for only the anterior left ventricle, due to high ATSM signal from the liver preventing further analysis. ATSM SUVs were quantified using data from the second postinjection hour.

### Tissue collection and immunohistochemistry

Normal control and 6-OHDA-treated rhesus macaques (at least 72 h after final PET) were euthanized by transaortic perfusion; hearts were processed for immunohistochemistry against tyrosine hydroxylase (TH; Immunostar, 1:400) and PGP9.5 (Millipore, 1:200).^13^ ImageJ was used to calculate percent area above threshold (%AAT) and optical density (OD)^13^ for six nerve bundles per region (septal, anterior, lateral, inferior) at three cardiac levels (base, middle, and apex).

### Statistical analysis

Statistical analysis of clinical measures was performed using GraphPad Prism version 5.0 f. A *p* < 0.05 was accepted as significant. Comparisons between time points within an individual treatment group were performed using repeated measures analysis of variance (ANOVA) with Bonferroni multiple comparison test correction. Comparison between treatments over time were done using two-way repeated measures ANOVA with the Huynh–Feldt correction for possible violations of sphericity.

Statistical analysis of PET data was conducted in R version 3.2.4 and SPSS version 23.0. DVs from MHED PET and SUVs from PBR28 PET at each time point were analyzed using ANOVA as 2 (Treatment: pioglitazone vs. placebo) × 8 (Regions: septal through inferior) × 6 (Level: base to apex) with repeated measures on region and level. Time (3: baseline, 1 week, 12 weeks) was included for analysis of change over time for PBR28 with pairwise comparison effect size calculated as Cohen’s *d*_*z*_.^60^ Huynh–Feldt adjusted *p* values were used for all effects involving repeated measures; Bonferroni correction was applied for multiple comparisons. Effects involving region and level at each time point were decomposed using standard polynomial trend analysis to identify relations to anatomy. Polynomial trend analysis results are reported as significance and as the percentage of variance in the effect that is accounted for by the polynomial trend component.^61^ Effect size is reported as partial eta squared (*η*_p_^*2*^), calculated in SPSS (Tables 1 and 2). Trends of order 4 or higher were disregarded unless they accounted for >10% of variance and residual tests from lower level trends were significant. Polynomial trend analysis describes the shapes of uptake for 48 anatomical areas (6 levels × 8 regions) of the cardiac left ventricle and avoids the use of individual statistical tests. Polynomial trend analysis is presented as surface plots in 3D space using the package “lattice” in R. Averages are mean ± SE unless otherwise specified. ATSM was analyzed by Mann−Whitney at each time point and Wilcoxon signed rank for change over time; effect size calculated as *r* = *Z*/(sqrt(*N*)). Immunohistochemistry data were analyzed using SPSS version 23.0. %AAT and OD data were analyzed using ANOVA as 3 (Treatment: pioglitazone vs. placebo vs. normal control) × 4 (Regions: septal through inferior) × 3 (Level: base, middle, apex) with repeated measures on region and level, post hoc analysis using Bonferroni multiple comparisons, and Huynh–Feldt adjusted *p* values for repeated measures. Pairwise effect sizes were calculated as Cohen’s *d*_*z*_ for within subject comparisons and Hedges’s *g*_*s*_ for between subject comparisons.^60^ Correlations between 12-week MHED and TH %AAT were performed following previously described methods of repeated measures correlations^62^ in R (v3.4.2).

### Data availability

The datasets generated during and/or analyzed during the current study are available from the corresponding author on reasonable request.

## Electronic supplementary material

Supplementary Material
