# Supplementary material for: In vivo imaging of inflammation and oxidative stress in a nonhuman primate model of cardiac sympathetic neurodegeneration
Source: NPJ Parkinsons Dis. 2018 Jul 13;4:22. doi: 10.1038/s41531-018-0057-1 (PMC6045637; doi:10.1038/s41531-018-0057-1)
Supplement: Supplementary file 1 — Supplementary Material [file 41531_2018_57_MOESM1_ESM.pdf]

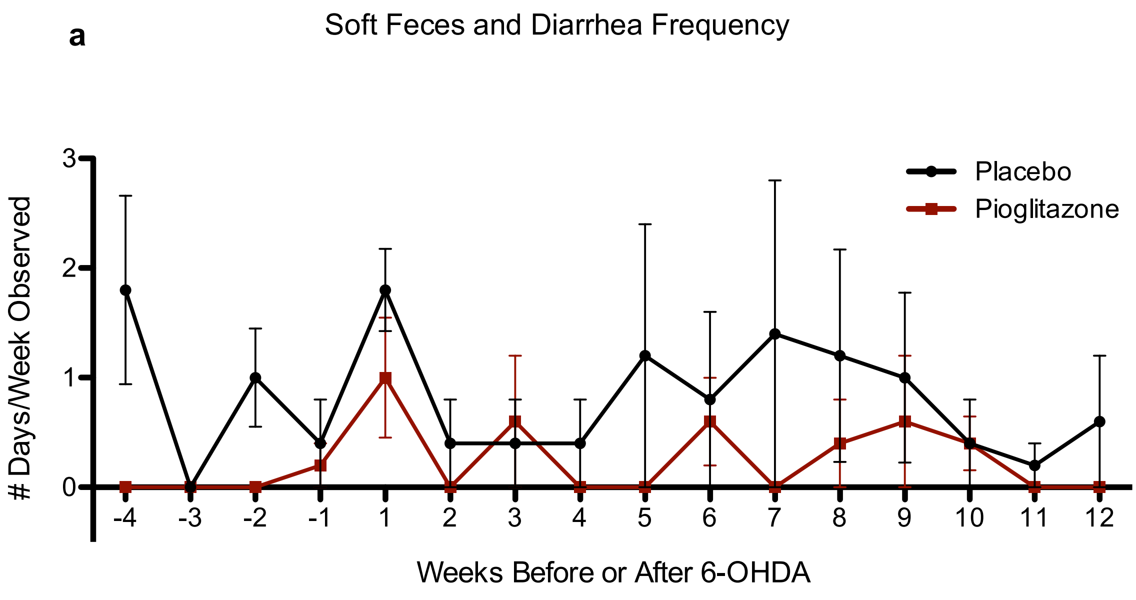

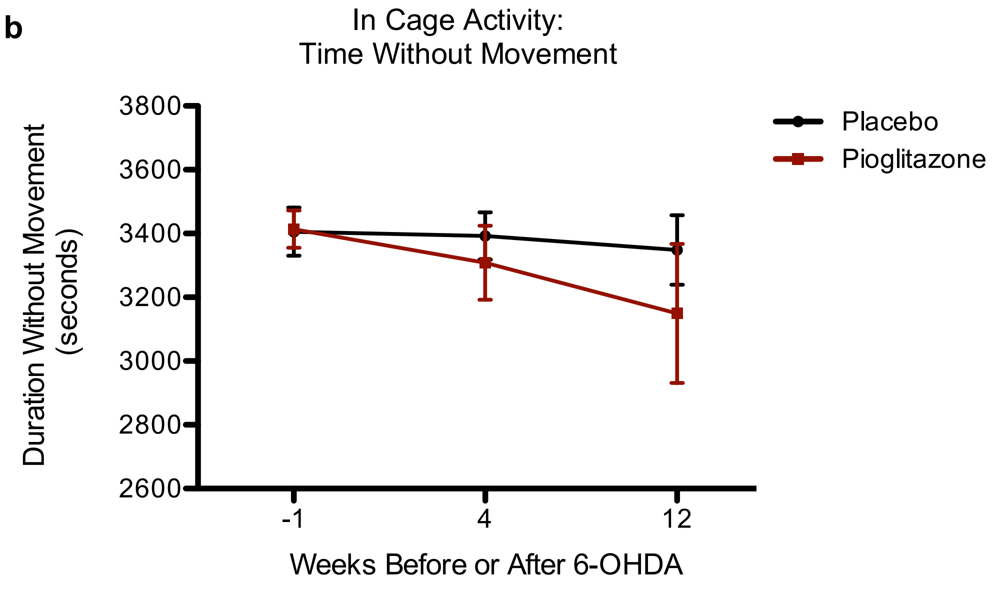

Supplementary Figure 1. **(a)** Total number of observations of either soft feces or diarrhea per week in placebo- and pioglitazone-treated animals. Note the increase in incidents in both groups immediately following 6-OHDA, which return to near baseline by week 2. **(b)** Average total time without movement for placebo- and pioglitazone-treated animals during in cage activity recording. No change was seen over time for either treatment group. Error bars ± SE.

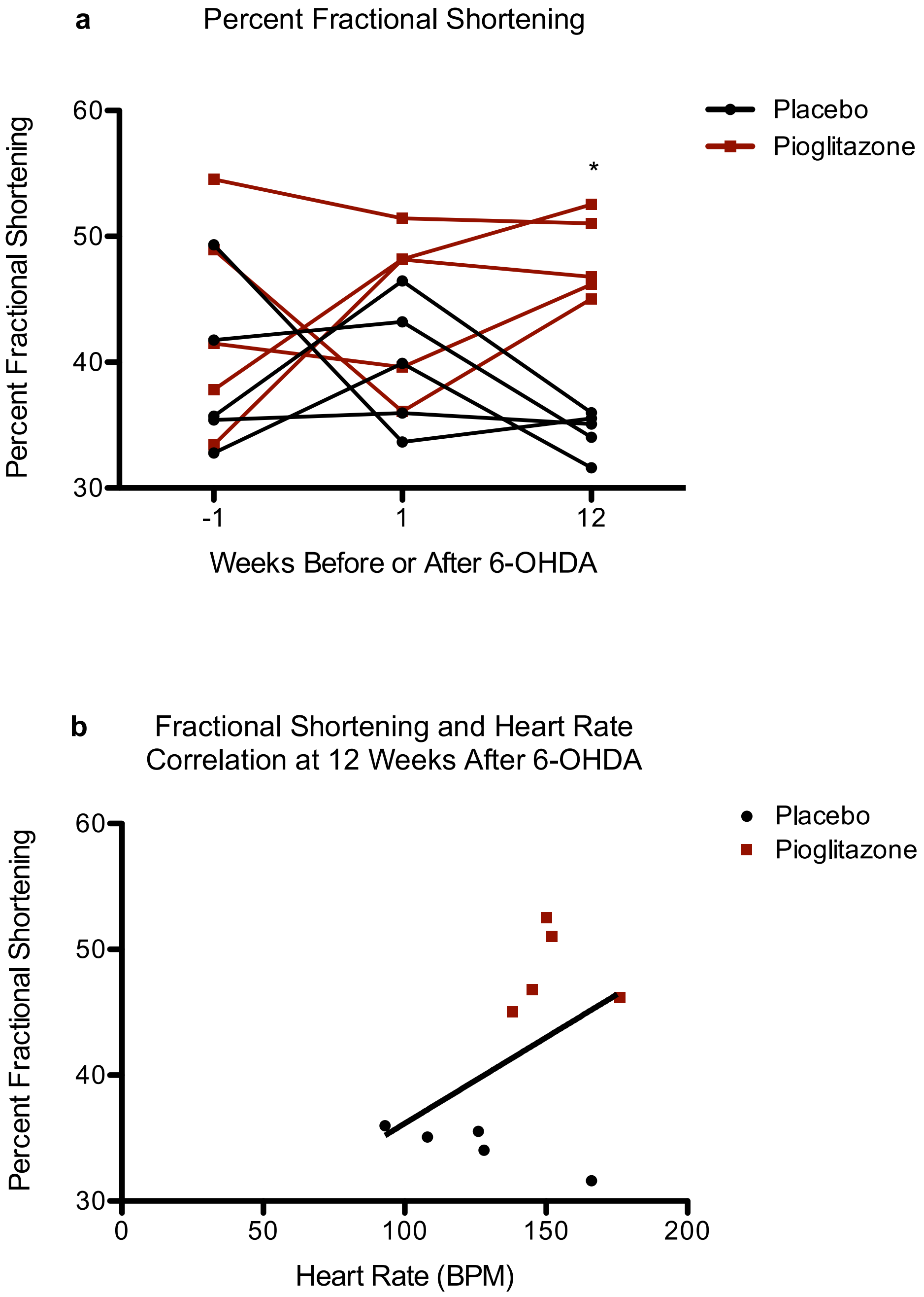

Supplementary Figure 2. **(a)** Percent fractional shortening (%FS) in placebo- and pioglitazone-treated animals. Each line represents an individual animal over time. At 12 weeks after 6-OHDA, %FS was significantly higher in pioglitazone-treated animals (48.32 ± 1.31) compared to placebo (34.45 ± 0.70) (*, $t(8)=8.4$, $p<0.0001$, $g_s=5.3$). **(b)** Relationship between %FS and heart rate at 12 weeks after 6-OHDA ($R^2=0.2$, $p>0.19$, 95% CI [-0.25, 0.84]). Each data point represents an individual animal.

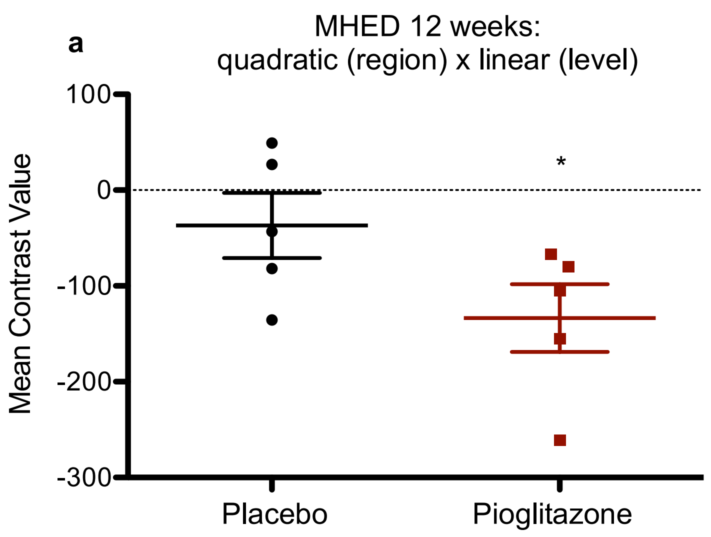

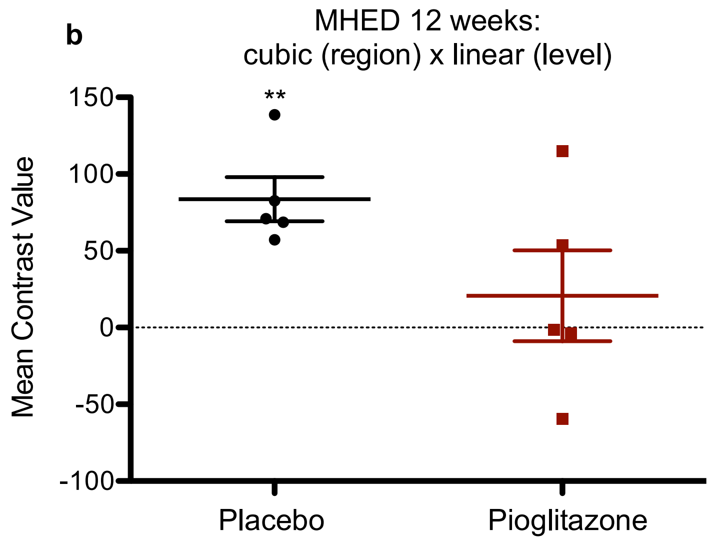

Supplementary Figure 3. Pioglitazone administration induced a unique distribution of cardiac MHED uptake at twelve weeks after 6-OHDA as detected by polynomial trend analysis. (a) Uptake in the pioglitazone-treated group displayed a quadratic (region) by linear (level) shape. Although this effect did not reach statistical significance between treatment groups ($t(8)=2.0$, $p>0.084$, $g_s=1.1$), the pioglitazone group was significantly different from zero (*, -133.5 ± 35.3; $t(4)=3.8$, $p<0.02$, $d=1.7$) while the placebo group was not (-36.8 ± 34.12; $t(4)=1.1$, $p>0.34$, $d=0.5$). **(b)** In contrast, the placebo-treated group exhibited a cubic by linear shape. This effect again did not reach statistical significance between treatment groups ($t(8)=1.9$, $p>0.092$, $g_s=1.1$); however, the placebo group was significantly different from zero (**, 83.6 ± 14.3; $t(4)=5.8$, $p<0.004$, $d=2.6$) while the pioglitazone group was not (20.77 ± 29.5; $t(4)=0.7$, $p>0.52$, $d=0.3$). Effect size for one sample t-test: $d= (\bar{x}-\mu_0)/s$. Error bars ± SE.

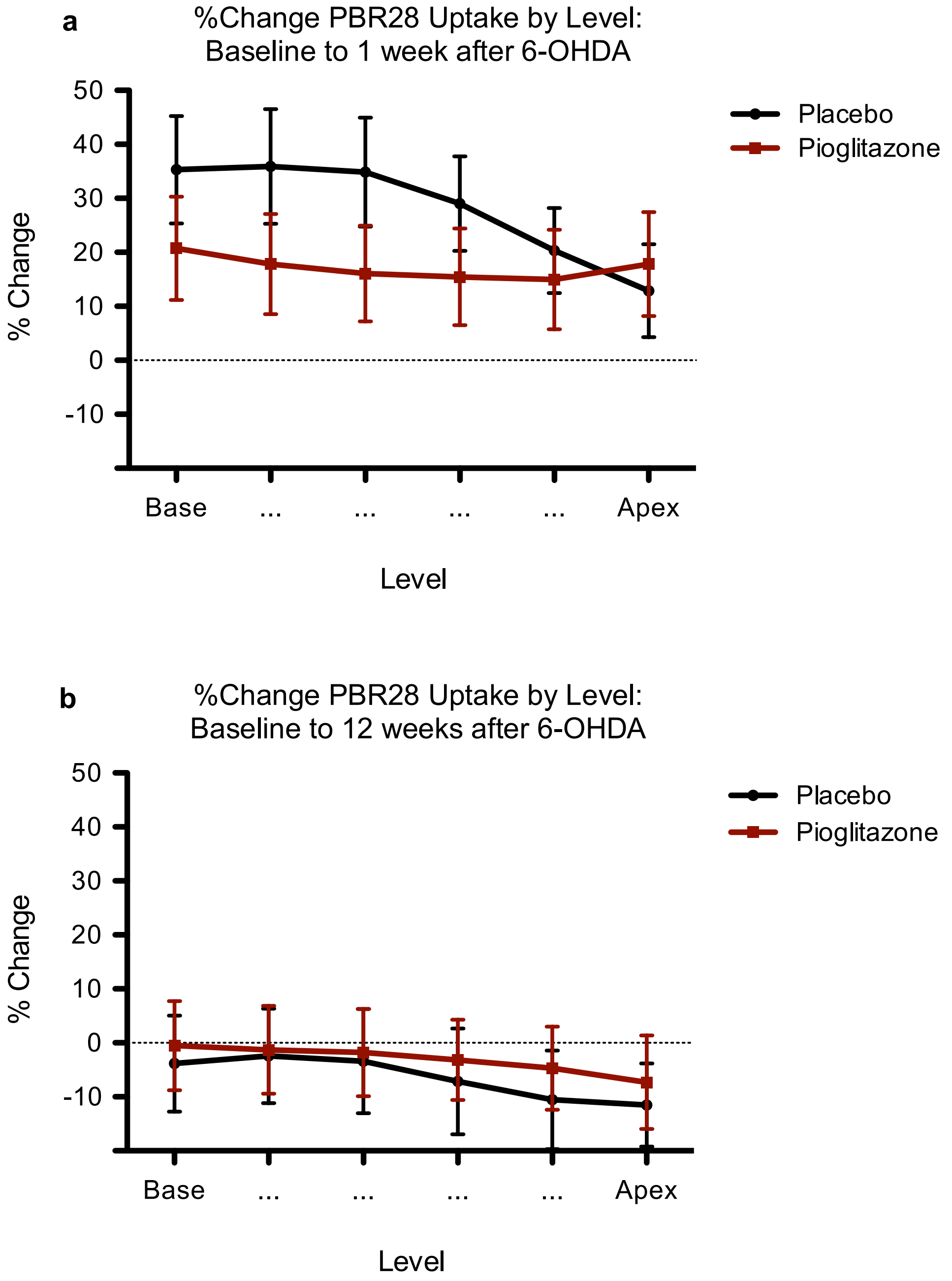

Supplementary Figure 4. Percent change of group average PBR28 uptake **(a)** from baseline to one week after 6-OHDA and **(b)** from baseline to 12 weeks after 6-OHDA in base to apex levels of the cardiac left ventricle. **(a)** Note the statistically significantly greater increase in PBR28 uptake in the placebo-treated group compared to pioglitazone at one week post-neurointoxication that was dependent on cardiac level (Condition x Level ANOVA $F(5,40)=7.50$, $p<0.008$, $\eta_p^2=0.5$). **(b)** By 12 weeks, animals in both groups returned to near baseline and no difference between groups was observed. Error bars ± SE.

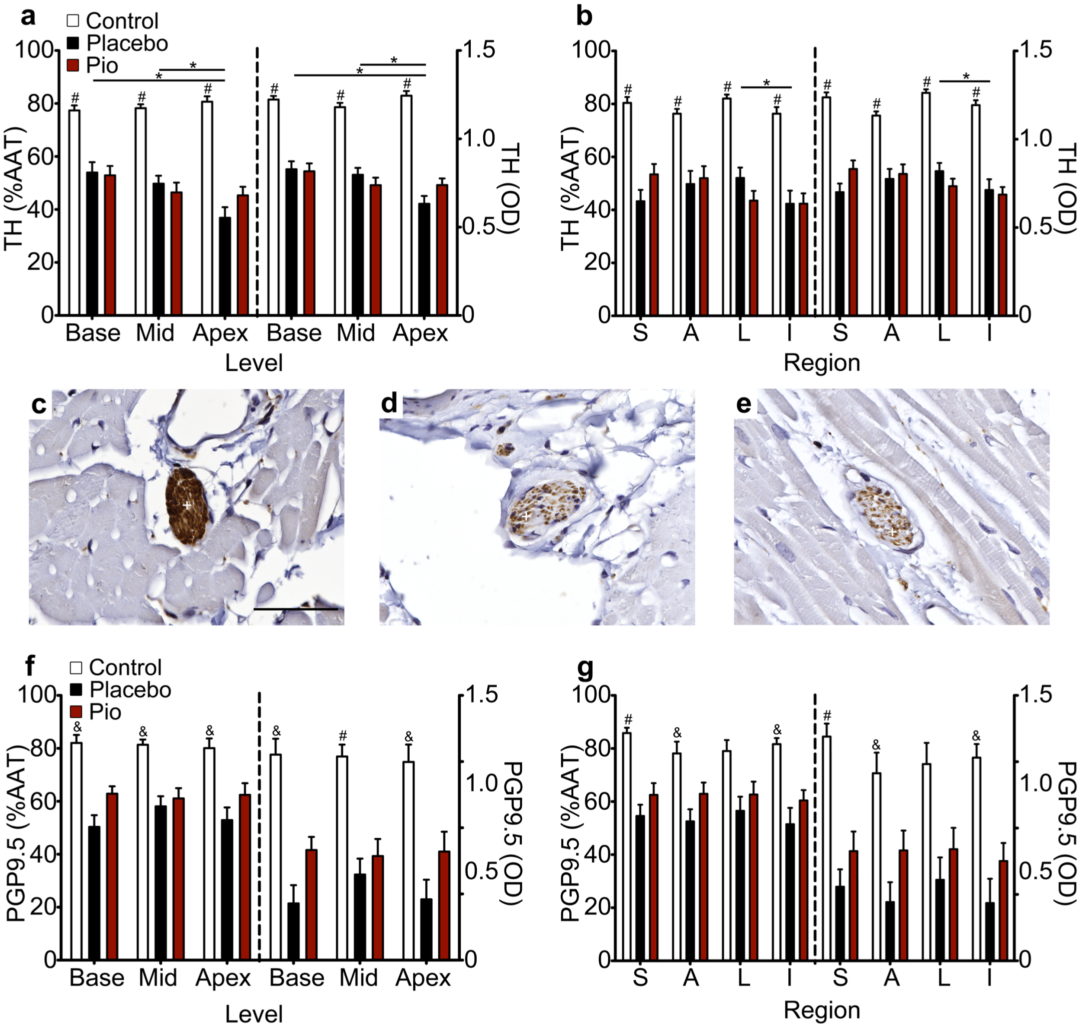

Supplementary Figure 5. Post mortem evaluation of the sympathetic innervation marker tyrosine hydroxylase (TH) and the panneuronal marker PGP9.5 throughout the cardiac left ventricle. (a) Across all cardiac levels, TH immunoreactivity (-ir) was significantly lower in placebo and pioglitazone groups compared to control. In placebo animals, the apex was significantly different from middle and base levels. **(b)** Across all cardiac regions, TH-ir was lower in placebo- and pioglitazone-treated animals compared to controls. In placebo animals, lateral and inferior regions were significantly different. When the entire left ventricle was averaged, TH-ir was significantly affected by treatment (%AAT $p<0.00007$; OD $p<0.00002$); both placebo (%AAT $p<0.0002$; OD $p<0.00004$) and pioglitazone (%AAT $p<0.0003$; OD $p<0.00005$) groups had significantly less TH-ir than controls. There were significant effects of cardiac anatomy in combination with treatment (Treatment x Region (%AAT $p<0.05$; OD $p<0.01$); Treatment x Level (%AAT $p<0.003$; OD $p<0.001$). **(c-e)** Photomicrographs of cardiac left ventricle sections immunostained against the panneuronal marker PGP9.5 (to validate neurodegeneration) and counterstained with hematoxylin. **(c)** Corresponds to control, **(d)** placebo- and **(e)** pioglitazone- treated monkeys. PGP9.5-ir was observed in bundles of nerves (white +), and in individual nerve fibers, similar to TH-ir (see main text Figure 4). **(f)** Across all cardiac levels PGP9.5-ir was significantly lower in placebo animals than controls; in pioglitazone animals the middle level was significantly lower compared to controls. **(g)** Regionally, control animals exhibited higher PGP9.5-ir compared to placebo in septal, anterior, and inferior regions, and higher PGP9.5-ir compared to pioglitazone in the septal region When the entire left ventricle was averaged, PGP9.5-ir was affected by treatment (%AAT $p<0.02$; OD $p<0.007$). Post hoc tests revealed that the placebo group was significantly different from controls (%AAT $p<0.02$; OD $p<0.007$), while pioglitazone was not (%AAT $p>0.08$; OD $p>0.05$). See Supplementary Table 5 for detailed statistical results. All %AAT and OD data were collected by two observers blind to treatment groups; interrater reliability showed high correlation between raters (%AAT ICC(3,2)=0.988; OD ICC(3,2)=0.985). Scale bar = 50µm. Error bars = SEM. #, control group significantly different from placebo and pioglitazone ($p<0.05$). &, control group significantly different from placebo ($p<0.05$). *, $p<0.05$. %AAT, percent area above threshold; OD, optical density; TH, tyrosine hydroxylase; PGP9.5, protein gene product 9.5.

Supplementary Table 1. (a) Animal weights (kg) at baseline, 1, and 11 weeks after 6-OHDA. A subset of animals experienced weight loss during the week following neurotoxin; weights returned to near baseline by 11 weeks. **(b)** Clinical rating scores for all animals at approximately one month after 6-OHDA. Note that systemic 6-OHDA did not induce motor dysfunction. R; right hand. L; left hand. Post.; posture. Bradykin.; generalized bradykinesia. Bal.; balance. DR; defense reaction. Freez.; freezing.

a

	Animal	Baseline Weight	1 Week Weight	11 Weeks Weight
Placebo	1	11.1	10.18	10.98
	2	10.6	10.07	9.8
	3	11.92	12	12.3
	4	8.92	9.06	10.7
	5	10.05	10	10.23
Pioglitazone	6	10.55	10.09	10.67
	7	9.07	8.6	9.9
	8	10.45	9.9	10.12
	9	9.13	9.67	10.1
	10	8.9	9.5	10.5

b

	Animal	Tremor R	Tremor L	Post.	Gait	Bradykin.	Bal.	Motor Skill R	Motor Skill L	DR	Freez.
Placebo	1	0	0	0	0	0	0	0	0	0	0
	2	0	0	0	0	0	0	0	0	0	0
	3	0	0	0	0	0	0	0	0	0	0
	4	0	0	0	0	0	0	0	0	0	0
	5	0	0	0	0	0	0	0	0	0	0
Pioglitazone	6	0	0	0	0	0	0	0	0	0	0
	7	0	0	0	0	0.5	0	0	0	0	0
	8	0	0	0	0	0	0	0	0	0	0
	9	0	0	0	0	0	0	0	0	0	0
	10	0	0	0	0	0	0	0	0	0	0

Supplementary Table 2. Troponin I values (ng/mL) for each animal at baseline, 1, 4, 8, and 12 weeks after 6-OHDA. Four placebo- and two pioglitazone-treated animals presented mild elevations in troponin one week after 6-OHDA that resolved by 12 weeks.

	Animal	Baseline Troponin	1 week Troponin	4 weeks Troponin	8 weeks Troponin	12 weeks Troponin
Placebo	1	0.012	0.657	0.012	0.016	0.012
	2	0.017	0.17	0.012	0.012	0.017
	3	0.012	0.151	0.031	0.026	0.026
	4	0.092	0.054	0.012	0.012	0.312
	5	0.013	0.288	0.022	0.015	0.015
Pioglitazone	6	0.018	0.2	0.012	0.022	0.012
	7	0.012	0.027	0.012	0.013	0.013
	8	0.014	0.028	0.012	0.013	0.057
	9	0.079	0.262	0.047	0.06	0.117
	10	0.017	0.079	0.013	0.012	0.016

Supplementary Table 3. Electrocardiogram (ECG) data from all animals at baseline, 1, and 12 weeks after 6-OHDA. No significant differences were found for any ECG values over time or between treatment groups. QTc was calculated using a rhesus specific QT120 correction as QT interval/sqrt (RR interval), where RR interval was calculated 120/HR due to the higher average resting heart rate in rhesus (120bmp) compared to humans (60bpm) (Hassimoto and Haranda, 2002). ΔHR, ΔPR, ΔQRS, and ΔQTc were calculated as the value for each time point minus the baseline value. HR, heart rate; PR, PR interval; QRS, QRS interval; QT, QT interval. ECG data indicated normal sinus rhythm, with the exception of one placebo-treated animal (#4), which exhibited sinus arrhythmia at all time points including baseline.

	Animal	Time	HR (bpm)	ΔHR	PR (ms)	ΔPR	QRS (ms)	ΔQRS	QT (ms)	ΔQTc
Placebo	1	baseline	130		102		42		240	
		1 wk	125	-5	118	16	39	-3	260	15.6
		12 wk	134	4	120	18	42	0	238	1.7
	2	baseline	102		83		42		284	
		1 wk	111	9	79	-4	39	-3	304	30.5
		12 wk	125	23	80	-3	40	-2	244	-12.8
	3	baseline	105		115		45		280	
		1 wk	108	3	108	-7	42	-3	300	22.7
		12 wk	105	0	120	5	40	-5	250	-28.1
	4	baseline	111		116		40		284	
		1 wk	110	-1	112	-4	44	4	288	2.6
		12 wk	109	-2	108	-8	41	1	284	-2.5
	5	baseline	135		118		42		210	
		1 wk	152	17	90	-28	40	-2	245	53.0
		12 wk	155	20	100	-18	40	-2	198	2.3
Pioglitazone	6	baseline	142		121		46		200	
		1 wk	115	-27	99	-22	42	-4	250	27.2
		12 wk	160	18	110	-11	40	-6	200	13.4
	7	baseline	130		110		40		242	
		1 wk	144	14	88	-22	48	8	240	11.0
		12 wk	180	50	90	-20	38	-2	202	-4.5
	8	baseline	135		102		43		220	
		1 wk	108	-27	80	-22	40	-3	250	3.8
		12 wk	146	11	101	-1	42	-1	220	9.3
	9	baseline	139		101		38		260	
		1 wk	130	-9	99	-2	42	4	250	-19.6
		12 wk	148	9	118	17	39	1	220	-35.5
	10	baseline	135		110		41		235	
		1 wk	138	3	84	-26	40	-1	250	18.8
		12 wk	160	25	82	-28	39	-2	200	-18.3

Supplementary Table 4. Echocardiogram data from all animals at baseline, 1, and 12 weeks after 6-OHDA. A 30% or larger increase of percent fractional shortening (%FS) that was sustained through 12 weeks after 6-OHDA occurred in two pioglitazone-treated monkeys, while %FS was decreased 27% in one placebo animal. Diastolic left ventricular diameter was decreased in the same two pioglitazone animals, while it increased in the placebo animal. All %FS values were within normal reference ranges (Heyen and Vargas, 2015); however, it should be noted that these ranges are for cynomolgus macaques, and publications are limited for rhesus. Group comparisons showed that at 12 weeks after 6-OHDA, %FS was significantly higher in pioglitazone- compared to placebo- treated monkeys ($p<0.05$; Supplementary Figure 3). %FS is calculated as [(LVDd-LVDs)/LVDd]x100. AWd, anterior wall thickness in diastole; AWs, anterior wall thickness in systole; PWd, posterior (inferior) wall thickness in diastole; PWs, posterior (inferior) wall thickness in systole. LVDd, left ventricle diameter in diastole; LVDs, left ventricle diameter in systole.

	Animal	Time	Awd (mm)	Aws (mm)	PWd (mm)	PWs (mm)	LVDd (mm)	LVDs (mm)	%FS	%FS: % change from baseline
Placebo	1	baseline	5.5	10.6	5.2	8.9	26.4	13.4	49.4	
		1 wk	5.4	9.3	5.1	8.1	29.4	19.5	33.7	-31.8
		12 wk	5.1	9.4	5.1	9.1	28.7	18.5	35.5	-28.0
	2	baseline	5.1	7.5	5.4	10.2	27.3	15.9	41.8	
		1 wk	5.2	12.0	5.2	6.2	27.8	15.8	43.2	3.5
		12 wk	5.0	7.8	4.9	7.7	30.0	19.8	34.1	-18.5
	3	baseline	5.1	9.4	5.1	9.9	28.9	18.6	35.4	
		1 wk	5.4	9.2	5.5	8.7	33.7	18.7	36.0	1.5
		12 wk	5.2	7.7	5.5	8.6	30.5	17.4	35.1	-0.9
	4	baseline	5.1	9.3	5.2	8.8	27.6	17.8	35.7	
		1 wk	5.4	10.8	5.2	8.6	25.0	13.4	46.5	30.2
		12 wk	5.3	7.7	5.3	8.2	25.9	16.6	36.0	0.8
	5	baseline	3.1	5.4	3.2	6.4	22.7	15.3	32.8	
		1 wk	3.4	7.4	3.4	7.0	22.9	13.8	39.9	21.8
		12 wk	3.9	8.2	3.6	7.6	24.4	16.7	31.6	-3.6
Pioglitazone	6	baseline	5.9	7.9	5.8	13.4	26.4	17.0	54.6	
		1 wk	5.7	10.8	5.4	10.3	27.0	13.1	51.4	-5.7
		12 wk	5.9	8.6	5.7	10.5	27.2	13.3	51.0	-6.4
	7	baseline	4.4	7.0	4.4	8.0	24.2	14.2	41.5	
		1 wk	4.4	7.7	4.4	9.3	27.3	16.5	39.6	-4.5
		12 wk	4.8	8.6	4.6	8.0	22.3	11.7	46.2	11.3
	8	baseline	5.7	9.6	5.4	11.4	28.2	14.4	48.9	-26.3
		1 wk	6.1	8.9	5.5	8.0	28.0	18.8	36.1	-8.0
		12 wk	6.2	9.3	5.8	9.7	28.4	15.6	45.0	
	9	baseline	4.9	8.5	4.9	8.6	27.6	18.4	33.4	
		1 wk	5.3	8.3	5.3	8.7	26.0	13.5	48.2	44.0
		12 wk	5.2	8.2	5.2	8.5	24.4	13.0	46.8	39.9
	10	baseline	4.9	9.0	4.8	8.6	25.5	15.8	37.8	
		1 wk	4.2	8.8	4.2	8.7	25.6	13.3	48.2	27.4
		12 wk	4.9	9.0	4.9	8.9	24.8	11.8	52.5	38.9

Supplementary Table 5. Results of repeated measures ANOVA analysis of post mortem immunohistochemical evaluation of sympathetic innervation (tyrosine hydroxylase; TH) and the panneuronal marker PGP9.5 illustrated in supplementary figure 5. (a) Significant pairwise comparisons of regions and levels within and between groups for TH. TH-ir was significantly affected by treatment (percent area above threshold (%AAT): $F(2,12)=25.1$, $p<0.00007$, $\eta_p^2=0.8$; optical density (OD): $F(2,12)=33.3$, $p<0.00002$, $\eta_p^2=0.8$). Both the placebo (%AAT: 46.86 ± 2.76, $t(12)=6.1$, $p<0.0002$, $g_s=2.1$; OD: 0.75 ± 0.03, $t(12)=7.1$, $p<0.00004$, $g_s=2.5$) and pioglitazone (%AAT: 48.22 ± 4.70, $t(12)=5.9$, $p<0.0003$, $g_s=2.2$; OD: 0.764 ± 0.056, $t(12)=6.9$, $p<0.00005$, $g_s=2.7$) groups had significantly less TH-ir than the controls (%AAT: 78.78 ± 1.66; OD 1.21 ± 0.03). There were significant effects of cardiac anatomy in combination with treatment (Treatment x Region (%AAT: $F(6,36)=2.4$, $p<0.05$, $\eta_p^2=0.3$; OD: $F(6,36)=3.6$, $p<0.01$, $\eta_p^2=0.4$); Treatment x Level (%AAT: $F(4,24)=5.6$, $p<0.003$, $\eta_p^2=0.5$; OD: $F(4,24)=6.9$, $p<0.001$, $\eta_p^2=0.5$). **(b)** Significant pairwise comparisons of regions and levels within and between groups for PGP9.5. Averaging over the left ventricle, PGP9.5-ir was affected by treatment (%AAT: $F(2,12)=6.8$, $p<0.02$, $\eta_p^2=0.5$; OD: $F(2,12)=8.0$, $p<0.007$, $\eta_p^2=0.6$). Post hoc tests revealed that the placebo group (%AAT: 53.78 ± 5.31; OD: 0.81 ± 0.06) was significantly different from controls (%AAT: 81.16 ± 4.03, $t(12)=3.6$, $p<0.02$, $g_s=1.5$; OD: 1.21 ± 0.06, $t(12)=3.9$, $p<0.007$, $g_s=1.6$), while pioglitazone was not (%AAT: 62.14 ± 5.35, $t(12)=2.5$, $p>0.08$, $g_s=1.2$; OD: 0.93 ± 0.08, $t(12)=2.7$, $p>0.05$, $g_s=1.2$). Although for PGP9.5 the effects of cardiac region or level in combination with treatment were not significant, pairwise comparisons were performed to identify specific anatomical areas contributing to the overall treatment effect. NR, not reported due to $p>0.05$.

a

TH Between Group Differences			TH Within Group Differences	
Comparison	Control v. Placebo	Control v. Pioglitazone	Comparison	Placebo
Base	%AAT $t(12)=4.1$, $p<0.005$, $g_s=1.6$<br>OD $t(12)=5.0$, $p<0.002$, $g_s=2.2$	%AAT $t(12)=6.0$, $p<0.004$, $g_s=1.8$<br>OD $t(12)=5.2$, $p<0.0008$, $g_s=2.3$	Base v. Apex	%AAT $t(8)=4.2$, $p<0.004$, $d_z=1.9$<br>OD $t(8)=4.3$, $p<0.004$, $d_z=1.9$
Middle	%AAT $t(12)=4.9$, $p<0.002$, $g_s=2.5$<br>OD $t(12)=4.6$, $p<0.0006$, $g_s=2.4$	%AAT $t(12)=6.7$, $p<0.0005$, $g_s=2.3$<br>OD $t(12)=5.3$, $p<0.0002$, $g_s=2.6$	Middle v. Apex	%AAT $t(8)=3.9$, $p<0.007$, $d_z=1.8$<br>OD $t(8)=4.7$, $p<0.002$, $d_z=2.1$
Apex	%AAT $t(12)=7.2$, $p<0.00004$, $g_s=2.9$<br>OD $t(12)=8.3$, $p<0.000002$, $g_s=3.5$	%AAT $t(12)=5.9$, $p<0.0003$, $g_s=2.7$<br>OD $t(12)=6.8$, $p<0.00002$, $g_s=3.2$	Lateral v. Inferior	%AAT $t(12)=3.2$, $p<0.046$, $d_z=1.4$<br>OD $t(12)=3.3$, $p<0.04$, $d_z=1.5$
Septal	%AAT $t(12)=6.2$, $p<0.0002$, $g_s=1.2$<br>OD $t(12)=6.9$, $p<0.00006$, $g_s=1.2$	%AAT $t(12)=5.9$, $p<0.003$, $g_s=1.1$<br>OD $t(12)=5.2$, $p<0.0007$, $g_s=1.2$		
Anterior	%AAT $t(12)=3.9$, $p<0.006$, $g_s=1.1$<br>OD $t(12)=4.3$, $p<0.003$, $g_s=1.1$	%AAT $t(12)=3.6$, $p<0.02$, $g_s=1.0$<br>OD $t(12)=4.0$, $p<0.006$, $g_s=1.1$		
Lateral	%AAT $t(12)=5.1$, $p<0.0009$, $g_s=1.1$<br>OD $t(12)=6.0$, $p<0.0002$, $g_s=1.2$	%AAT $t(12)=6.2$, $p<0.0002$, $g_s=1.2$<br>OD $t(12)=7.1$, $p<0.00004$, $g_s=1.2$		
Inferior	%AAT $t(12)=5.7$, $p<0.0003$, $g_s=1.1$<br>OD $t(12)=7.6$, $p<0.00002$, $g_s=1.2$	%AAT $t(12)=5.7$, $p<0.0003$, $g_s=1.2$<br>OD $t(12)=8.0$, $p<0.00002$, $g_s=1.2$		

b

PGP9.5 Between Group Differences		
Comparison	Control v. Placebo	Control v .Pioglitazone
Base	%AAT: $t(12)=3.3$, $p<0.006$, $g_s=1.7$<br>OD: $t(12)=4.1$, $p<0.005$, $g_s=1.8$	NR
Middle	%AAT: $t(12)=3.1$, $p<0.03$, $g_s=1.6$<br>OD: $t(12)=3.4$, $p<0.02$, $g_s=1.7$	OD: $t(12)=2.9$, $p<0.05$, $g_s=1.4$
Apex	%AAT: $t(12)=2.8$, $p<0.05$, $g_s=1.3$<br>OD: $t(12)=3.2$, $p<0.03$, $g_s=1.5$	NR
Septal	%AAT: $t(12)=4.2$, $p<0.004$, $g_s=1.1$<br>OD: $t(12)=4.3$, $p<0.003$, $g_s=1.2$	%AAT: $t(12)=3.1$, $p<0.03$, $g_s=1.0$<br>OD: $t(12)=3.3$, $p<0.02$, $g_s=1.1$
Anterior	%AAT: $t(12)=3.1$, $p<0.03$, $g_s=1.1$<br>OD: $t(12)=3.2$, $p<0.03$, $g_s=1.1$	NR
Lateral	NR	NR
Inferior	%AAT: $t(12)=3.5$, $p<0.02$, $g_s=1.0$<br>OD: $t(12)=3.9$, $p<0.007$, $g_s=1.1$	NR
